# Exome Analysis of Rare and Common Variants within the NOD Signaling Pathway

**DOI:** 10.1038/srep46454

**Published:** 2017-04-19

**Authors:** Gaia Andreoletti, Valentina Shakhnovich, Kathy Christenson, Tracy Coelho, Rachel Haggarty, Nadeem A Afzal, Akshay Batra, Britt-Sabina Petersen, Matthew Mort, R. Mark Beattie, Sarah Ennis

**Affiliations:** 1 Human Genetics & Genomic Medicine, University of Southampton, Duthie Building (Mailpoint 808), Southampton General Hospital, Southampton, SO16 6YD, UK; 2 The Children’s Mercy Hospital, Division of Gastroenterology, Hepatology and Nutrition, Kansas City, MO, USA; 3 The Children’s Mercy Hospital, Division of Clinical Pharmacology, Toxicology and Therapeutic Innovation, Kansas City, MO, USA; 4 Southampton Children’s Hospital, University Hospital Southampton NHS Foundation Trust, Southampton General Hospital, Tremona Road, Southampton, SO16 6YD, UK; 5 NIHR Nutrition Biomedical Research Centre, Southampton Centre for Biomedical Research, University Hospital Southampton NHS Foundation Trust (Mailpoint 218), Southampton General Hospital, Southampton, SO16 6YD, UK; 6 Institute of Clinical Molecular Biology, Christian-Albrechts-University of Kiel, University Hospital, Schleswig-Holstein, Schittenhelmstr 12, 24105 Kiel, Germany; 7 Cardiff University School of Medicine, Institute of Medical Genetics Building, Heath Park, Cardiff CF14 4XN, UK

## Abstract

Pediatric inflammatory bowel disease (pIBD) is a chronic heterogeneous disorder. This study looks at the burden of common and rare coding mutations within 41 genes comprising the NOD signaling pathway in pIBD patients. 136 pIBD and 106 control samples underwent whole-exome sequencing. We compared the burden of common, rare and private mutation between these two groups using the SKAT-O test. An independent replication cohort of 33 cases and 111 controls was used to validate significant findings. We observed variation in 40 of 41 genes comprising the NOD signaling pathway. Four genes were significantly associated with disease in the discovery cohort (*BIRC2* p = 0.004, *NFKB1* p =  0.005, *NOD2* p = 0.029 and *SUGT1* p = 0.047). Statistical significance was replicated for BIRC2 (p = 0.041) and *NOD2* (p = 0.045) in an independent validation cohort. A gene based test on the combined discovery and replication cohort confirmed association for *BIRC2* (p = 0.030). We successfully applied burden of mutation testing that jointly assesses common and rare variants, identifying two previously implicated genes (*NFKB1* and *NOD2*) and confirmed a possible role in disease risk in a previously unreported gene (*BIRC2*). The identification of this novel gene provides a wider role for the inhibitor of apoptosis gene family in IBD pathogenesis.

Inflammatory bowel disease (IBD) is an umbrella term for a group of complex and multifactorial illnesses: Crohn’s disease (CD), ulcerative colitis (UC) and inflammatory bowel disease unclassified (IBDU)^1^. The etiology of IBD is multi-genic and environmentally triggered, but generally accepted to occur as a result of an inappropriate immune response to the normal gut flora in genetically predisposed individuals^2^.

Since the discovery of *NOD2* in 2001 as the first susceptibility gene for IBD^3^, over 200 loci have been associated with IBD risk in humans through genome wide association studies (GWAS)^4^,^5^. GWAS have provided substantial insight into the understanding of the biology of complex diseases by providing robust and replicated evidence for autophagy^2^, immune response^2^ and bacterial recognition^2^ patterns. However, an intrinsic limitation of these studies is their focus on common variation, typically those with a minor allele frequency (MAF) ≥5% in the general population. The combined contribution of these common mutations to IBD heritability only account for 13.6% of CD and 8.2% of UC, respectively^6^. It is hypothesized that low frequency (MAF of 0.05–5%) and rare (MAF ≤ 0.05%) variation may contribute significantly towards some fraction of the missing heritability of IBD^6^,^7^,^8^.

Recent technological advances in DNA sequencing have made it possible to sequence large tracts of the genome in a cost-effective manner. This has enabled large-scale studies of the impact of rare variants on complex diseases^9^. Whole-exome (WES) and whole genome sequencing (WGS) have improved the understanding of genetic cause of diseases by revealing variants not captured by GWAS^10^. It is estimated that ~85% of disease-causing mutations reside within the coding regions of the genome^11^. Therefore, targeting these expressed regions of the genome represents the most cost-effective means to uncover causal disease genes^12^.

Pediatric onset IBD (pIBD) presents with unique phenotypic characteristics and pronounced severity compared to adult-onset disease^13^. PIBD is more often characterized by extensive intestinal involvement, rapid early progression and a high rate of resistance to conventional therapy^1^. Moreover, early-onset IBD has a stronger familial component than adult disease^1^. These combined features indicate a stronger genetic component to pIBD compared to IBD diagnosed in adulthood.

GWAS are powered to assess common genetic variation in large patient cohorts that are often composed of adults, in order to amass sizeable patient groups. Large cohorts of patients with disease onset in childhood are less easily ascertained and also likely enriched for rare or private variation of large effect^14^. Approximately 300 genes have been prioritized within the 200 loci determined through adult studies and only less than half have been replicated in a small number of pediatric studies^15^,^16^.

To date, 51 genes have been associated with monogenic disease manifesting in an early onset IBD-like phenotype^17^,^18^. Homozygous mutations in the interleukin 10 receptor (*IL10*) gene and its associated receptor alpha and beta subunits (*IL10RA* and *IL10RB*) have been associated with children presenting very-early-onset IBD (VEO, age of onset <6 years)^19^,^20^. The discovery of the disease causal mutations helped to personalize treatments inducing a sustained remission in the patients^19^,^20^. The investigation of a child with intractable IBD using whole-exome analysis by Worthey *et al*.^21^ found a hemizygous mutation in the gene X-linked inhibitor of apoptosis (*XIAP*). The same mutation was confirmed in the asymptomatic mother. Based on these findings, this patient underwent hematopoietic stem cell progenitor transplantation with a resolution of symptoms and sustained remission following this targeted treatment approach^21^.

*XIAP* belongs to the in inhibitor of apoptosis protein (IAP) family (comprising *XIAP, BIRC2* and *BIRC3*) that plays a role in regulation of the innate immune response through ubiquitin ligase activity, TNF survival, inflammatory and death signaling pathways^22^,^23^. IAP proteins mediate the downstream signaling of pattern-recognition receptors such as NOD1 and NOD2 after response to bacterial pathogens^24^.

The NOD signaling pathway, Fig. 1, is involved in gram-negative and gram-positive peptidoglycan recognition. NOD1 and NOD2 proteins are highly conserved cytoplasmatic receptors that sense microbial effectors. Activation of NOD receptors leads to downstream activation of multiple molecules including mitogen-activated protein kinases (MAPK) and nuclear factor kappa-light-chain-enhancer of activated B cells (NF-κB)^25^.

In this study, we hypothesize that rare and private genetic variation across genes involved in the NOD signaling pathway may contribute to childhood onset IBD. We interrogate WES data to extract all genetic variation across the frequency spectrum in a pIBD cohort and evaluate the joint effect of rare and common variants with a gene-based statistical test (SKAT-O^26^). We further validate our findings in an independent cohort.

## Results

PCA procedure removed 10 cases and 20 controls reducing the final number of cases to 136 and controls to 106 within the discovery cohort (Supplementary Figure 1). The analysis revealed no outliers in the replication cohort. (Supplementary Figures 2 and 3).

Mutations were identified in either cases and/or controls in all but one gene (*CCL5*) from the NOD signaling pathway in the discovery cohort (n_cases_ = 136 and n_controls_ = 106). A total of 250 variants (Supplementary Table 2) that occurred in at least one individual (either case or control) across 41 genes were called in order to extract and create the VCF file for all 242 individuals. We observed 67 novel variants, 94 rare variants with a MAF 1000 genome project _(1 KG)_ <1%, 41 low frequency mutations (1% ≤ MAF_1 KG_ ≥ 5%) and 48 common mutations (MAF_1 KG_ > 5%). The majority of these variants would not have been detected or interrogated using array technology or traditional association studies.

### Variants within the NOD2 gene

**Figure 2:**
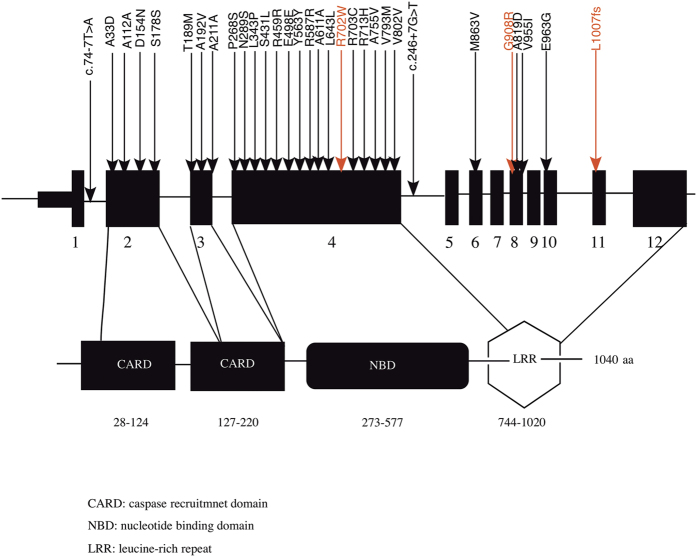
NOD2 gene and protein. NOD2 is a gene composed of 12 exons (black rectangles). The NOD2 protein consists of two N-terminal caspase activation recruitment (CARD) domains, a central nucleotide-binding oligomerization (NBD) domain and a terminal sequence rich in leucine (LRR). The CARD domains interact with RIP2 protein to activate the immune response in the gut and the leucine-rich domain recognizes the bacterial peptidoglycan. Mutations within the NBD have been shown to increase the inflammatory cascade. The 31 mutations observed by interrogating exome data from 136 pIBD and 106 controls are depicted using arrows and the corresponding protein change noted. Known IBD biomarkers are in red.

Across the 126 pIBD cases and 85 controls within the discovery cohort, we observed 31 mutations over 12 exons of the *NOD2* gene. Of these, 26 had a MAF <0.05 across the cohort (Table 1). Eight mutations were identified in or proximal to the caspase recruitment (CARD) domain, 16 in the nucleotide-binding oligomerization (NBD) domain and seven in the leucine-rich (LRR) domain (Fig. 2). In addition to the known IBD biomarkers, Arg702Trp, Gly908Arg and Leu1007fsinsC^3^,^27^, we observed two novel variants, 20 rare (MAF_1 KG_ < 0.01), two low frequency (0.01 ≤ MAF_1 KG_ ≤ 0.05) and four common mutations (MAF_1 KG_ > 0.05) (Table 1). Ten of the 26 mutations were annotated as deleterious by SIFT and 13 are described in HGMD as pathogenic^28^. Twenty six (out of 31) mutations observed would not have been assessed in any GWAS due to their rarity.

### Gene based burden of mutation testing in the discovery cohort

The gene-based test for assessing the combined association of novel, rare and common mutation with disease status showed significant evidence for association with four genes across the discovery cohort (*BIRC2, NFKB1, NOD2*, and *SUGT1* see Table 2). *NFKB1* (p = 0.005) and *NOD2* (p = 0.029) are known IBD associated genes. *SUGT1* is a previously unreported gene but has borderline significance only (p = 0.047). Combined variation in BIRC2 is more significantly associated (p = 0.004) with IBD in our discovery cohort than any other genes. This gene has not been previously implicated by association studies.

### Replication of the gene based burden of mutation test in the validation cohort

We aim to conduct a replication analysis of the four gene identified as significant in the discovery phase using a replication cohort (n_cases_ = 33; n_controls_ = 111). A total of 13 variants were identified across the regions sequenced in the *NFKB1, BIRC2, NOD2* gene. No variant was observed in *SUGT1* in the replication cohort and therefore SKAT-O test was not conducted on this gene. SKAT-O test showed independent statistical association for *BIRC2* (p = 0.041) and *NOD2* (p = 0.045) but was not powered to detect significant association for *NFKB1* (p = 0.223), Table 3. The gene based test on the combined discovery and replication cohort (n cases  =  169 and n controls  =  217) showed statistical association for *NOD2* (p = 0.011), NFKB1 (p = 0.017) and *BIRC2* (p =  0.030), Table 3.

## Discussion

Since 2005 next generation sequencing (NGS) has proven to be an effective technology for the study of rare and low frequency mutations within disease-associated genes^29^. More than 100 types of Mendelian disorders have been studied using WES with a diagnostic rate of success of 25–30%^30^. This success represents a substantially higher rate than that afforded by classical clinical genetic testing such as karyotyping (<5%) or array comparative genomic hybridization (~15–20%)^30^ The combination of traditional genetic testing and WES/WGS technology has rapidly accelerated the discovery of new disease-associated genes underlying Mendelian traits: from an average of 166 per year between 2005^30^ and 2009 to 236 per year between 2010 and 2014^30^, with the numbers increasing every year. WES/WGS has made gene discovery for all phenotypes feasible and cost effective^30^. The rapid growth and success of the next generation sequencing technologies in Mendelian traits has brought a great interest in their application to complex traits. WES and WGS have enable diagnosis and alternative treatment in patients with monogenic IBD^18^.

In our study we applied WES and the SKAT-O statistical test on a discovery cohort of 242 individuals. We conducted the analysis with no assumption with regard to IBD diagnosis (CD, UC or IBDU) because in half of the families recruited in the study we observed mixed diagnoses reflecting the substantial genetic overlap between IBD subtypes. Although our data were derived from whole exome sequencing, we did not conduct SKAT-O on all gene across the exome due to our modest sample size. Instead, we targeted our analysis to all 41 genes across the NOD signaling pathway removing the requirement of an exome-wide significance threshold^31^. We chose to select the most significantly associated genes and to replicate their significance in an independent replication cohort. A limitation of the replication analysis was the use of data gleaned from different sources. Although an established method to take into account such differences is not yet available^32^,^33^, we minimized bias by analyzing only variants that occurred in the regions common to all capture kits.

Despite a modest cohort size, we detected significant association in four genes and replicated significant association for two genes (*NOD2* and *BIRC2*).

*NOD2* is the earliest gene implicated in IBD pathogenesis and the most strongly associated in association studies with IBD^34^. Polymorphisms within *NOD2* are known to increase the risk of developing CD.^35^ *NOD2* patient carriers of one of the three allelic biomarker variants have an increased risk of developing CD: heterozygous carriers have a 2–4-fold increased risk of CD, while homozygous or compound heterozygous carriers have a 20–40-fold increased risk^34^.The association for *NOD2* was solely driven by the three known biomarkers (Table S3).

**Figure 3:**
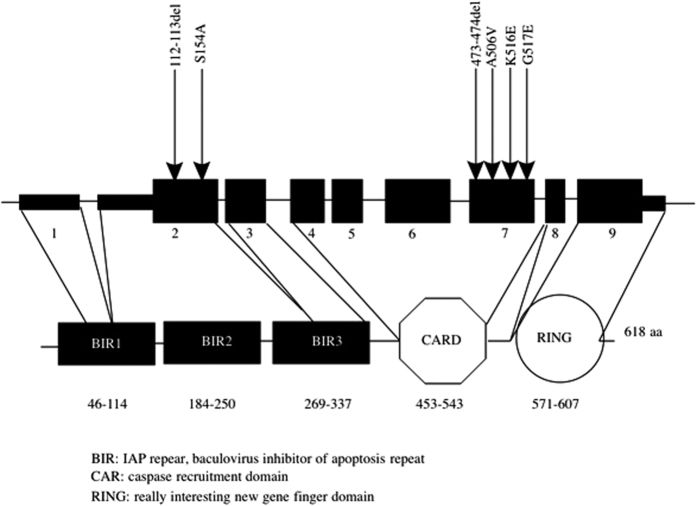
BIRC2 gene and protein. BIRC2 is a gene composed of 9 exons (black rectangles). BIRC2 encodes an inhibitor of apoptosis protein, which contributes to innate immune responses by acting as inhibitor of cell death. All the members of the inhibitor of apoptosis (IAP) gene family share three tandem specific motifs: BIR belonging to the zinc-finger domain that mediates protein-protein interaction, a CARD domain is involved in CARD-CARD mediated interaction; and a C-terminal RING domain conferring an E3-ubiquitin ligase activity. The RING domain of BIRC2, BIRC3, and XIAP is required for the ubiquitin activity of the IAPs. Studies have reported that the CARD domain in BIRC2 and BIRC3 act as an inhibitor of the ubiquitin ligase activity. Mutations within the BIR1 domain in *BIRC2* alters molecular interaction with TNF receptor associated factor 1 (TRAF1) and TRAF2. The six mutations found by interrogating exome data from 136 pIBD and 106 controls are depicted using arrows and the corresponding protein change shown.

*BIRC2* (Fig. 3) belongs to a gene family (*XIAP, BIRC2* (also known as cIAP1) and *BIRC3* (also known as cIAP2)) encoding three conserved proteins characterized by the presence of 1-3 baculovirus IAP repeat (BIR) motifs^36^. *XIAP* is located on the X chromosome while *BIRC2* and *BIRC3* are both located on chromosome 11. Several studies have demonstrated the importance of these genes in regulating the expression of proinflammatory cytokines, such as TNFα, through NF-kB and MAPK pathways primarily through their ubiquitin-ligase activity. *XIAP, BIRC2* and *BIRC3* are key players in regulating the NOD1 and NOD2 signaling pathway by directly promoting RIPK2 ubiquitylation and they facilitate activation of NF-kB pathway to promote cell survival^37^. Cellular studies on *BIRC2, BIRC3* and *XIAP* deficient macrophages were defective for MAPKs and NF-kB activation^23^,^38^. This defect in the NOD signaling was also further observed *in vivo* in *BIRC2, BIRC3* and *XIAP* knockout murine IBD models^38^. *BIRC2* and *BIRC3* are inhibitors of the Fas signaling cascade in human intestinal cell line^23^. The expression profile of *BIRC3* was further investigated in 14 UC patients indicating an overexpression in colonic specimens during disease flares^39^. Additional studies on the interleukin (IL)-11 expression suggested a possible protective role of IAP, indicating that an over-expression of the IAP proteins could promote healing of the gut^40^. It is therefore feasible that mutations within these genes might impact gut healing and contribute to flares in IBD. Six variants within *BIRC2* were observed in the discovery cohort across 15 cases and 4 controls. Three of these were novel (p.112_113del, p.S154A and p.G517E), two were rare (p.K516E and p.S318S) and one was low frequency (p.A506V,). Across the 15 cases (four with CD, four with IBDU and seven with UC), four were diagnosed aged <6 years, seven had a positive family history for IBD and nine were diagnosed with a second autoimmune condition other than IBD. While our observed enrichment of variation within *BIRC2* directly implicates this gene in pediatric IBD, further functional analyses are necessary for a comprehensive understanding of the role of individual variants in this protein and their wider impact on the signaling pathway. While mutations in *XIAP* are known to cause up to 4% of male early onset IBD, it is has been postulated that *BIRC2* and *BIRC3* might contribute to IBD pathogenesis by regulating the inflammatory cascade through their ubiquitin-ligase activity, our findings are the first to directly implicate this genes in pIBD^41^.

Novel drugs that mimic the natural endogenous inhibitor of the IAP (the mitochondria-derived activator of caspases, SMAC) have been proposed to suppress the pro-inflammatory immune response in the gastro-intestinal tract for patient with moderate to severe disease activity^42^. It is possible that patients harboring *BIRC2* mutations may benefit from new treatments targeting IAP expression and function. Further studies are required to assess the role of targeted therapy in the clinical management of these patients.

## Conclusions

A gene based burden of mutation test for association using sequencing data on a small cohort have supported the involvement of *NFKB1* and *NOD2* in the pathogenesis of IBD and have confirmed a role for *BIRC2* in the pathogenesis of disease. This is the first study highlighting the role of *BIRC2* in IBD through targeted exome sequencing.

## Methods

### Ethics statement

This study was approved by the Southampton and South West Hampshire Research Ethics Committee (REC) (09/H0504/125) and University Hospital Southampton Foundation Trust Research & Development (RHM CHI0497).

This study was approved by the Institutional Review Board (IRB) at The Children’s Mercy Hospital (IRB #15050179).

All methods were conducted in accordance with the relevant guidelines and regulations. Written informed consent was obtained for every participant.

### Cases and samples

For the discovery cohort, patients were recruited through pediatric gastroenterology clinics at University Hospital Southampton (UHS), a regional center providing tertiary pediatric gastroenterology and endoscopy service for the Wessex region in Southern England. Written informed consent was provided by an attending parent or legal guardian for all pediatric recruits. All children aged <18 at the point of diagnosis were eligible for recruitment to the study. The mean age of the cohort was 10.97 years (min 1–max 17 years). Diagnosis was established using the Porto criteria. Clinical data were recorded for each patient including family history of IBD and any history of autoimmune disease. We accessed control samples through our local database of germline exome sequence data for 126 unrelated patients with no inflammatory-related disease.

We used an independent replication cohort derived from the Children’s Mercy Kansas City IBD cohort and the Critical Assessment of Genome Interpretation (CAGI, 2013)^43^ dataset to validate significant results from the discovery cohort. The Children’s Mercy Kansas City cohort consists of 43 whole-exome individuals of which 13 are independent IBD patients and 1 control; the CAGI dataset is composed of 66 whole-exomes datasets (in VCF format) of which 20 are unrelated adult CD patients and 8 are unrelated healthy controls. We merged 102 additional whole-genome control samples of British ethnicity from the 1 KG phase 3 dataset^44^ resulting in the retention of 33 unrelated cases and 111 independent controls for subsequent analysis in the validation cohort.

### Discovery cohort DNA extraction

Genomic DNA for each of the Southampton patients undergoing exome sequencing was extracted from saliva or peripheral venous blood samples collected in EDTA using the salting out method. DNA concentration was estimated using the Qubit 2.0 Fluorometer and α260:280 ratio calculated using a nanodrop spectrophomter. The average DNA yield obtained was 150 μg/ml and approximately 20 ug of each patient DNA was extracted for next generation sequencing.

### Whole-exome sequencing data generation and analysis

For the Southampton discovery and Children’s Mercy Kansas City cohort, whole-exome capture was performed using Agilent SureSelect Human all Exon 51 Mb (versions 4 and 5) capture kits and TruSeq Expanded Exome and Nextera Expanded Exome capture kits. Capture technology is characterized by rapid progress, including new content and improved probe design, and we applied the optimal capture chemistry available at the time of sample sequencing. All samples were sequenced on the Illumina HiSeq 2000 and HiSeq 2500 platforms. As previously described^45^, fastQ raw data generated from Illumina paired-end sequencing protocol were aligned against the human genome reference 19 using Novoalign (2.08.02). SAMtools mpileup tool (samtools/0.1.19) to call SNPs and short indels. Variants called with a read depth <4 were excluded. The Phred software reads DNA sequencing trace files, calls bases, and assigns a quality value to each called base and is powered to discriminate between correct and incorrect base-calls. To minimize the false positive rate for the called bases, only variants called with high confidence (Phred score >20) were retained for further analysis (99% base call accuracy). ANNOVAR (annovar/2013Feb21) was then applied for variant annotation. Genetic variants were annotated as “novel” if they were not previously reported in the dbSNP137 databases, 1000 Genomes Project (1 KG) and the Exome Variant Server (EVS) of European Americans of the NHLI-ESP project with 6500 exomes, or in the Southampton database of reference exomes. Resultant variant call files for each individual were subjected to further in-house quality control tests to detect DNA sample contamination and ensure sex concordance by assessing autosomal and X chromosome heterozygosity. Variant sharing between all pairs of individuals was assessed to confirm that subjects were not related. Sample provenance was confirmed by application of a validated SNP tracking panel developed specifically for exome data^46^.

For the CAGI subgroup of the replication cohort, whole-exome sequencing was performed using the TruSeq capture kit and sequenced on Illumina platforms. Alignment against the human genome (hg19) was conducted with BWA. PICARD was used to remove duplicate reads and GATK for genotype calling. The VQSR method was used to identify true polymorphisms in the samples rather than those due to sequencing, alignment, or data processing artefacts^43^.

### Gene selection

Genes involved in the NOD receptor pathway were extracted by interrogating the KEGG Pathway database^47^. The pathway (KEGG ID: hsa04621) is composed of 56 genes, of which 41 are intrinsic to NOD signaling. Gene names were cross-referenced with the HUGO webserver to confirm the approved gene symbol (Supplementary Table 1). All good quality (Depth ≥ 4 and Phred ≥ 20) variants within these genes were extracted using local scripts and retained for analyses. SKAT-O statistical test was then performed on the 41 genes directly involved in the *NOD1* and *NOD2* signaling cascade.

### Principle component analysis

Whole-exome sequencing data were available for 146 independent children diagnosed with IBD within the discovery cohort. Demographic data for the IBD cohort are shown in Table 4.

In order to minimize bias for association analysis, we conducted a principle component analysis (PCA) using the SNPRelate^48^ package on the discovery and validation cohort to exclude non-Caucasian samples. PCA was conducted on the whole discovery dataset merged with the 1,092 subjects from the 1,000 genome phase 1 dataset (20101123) in order to discriminate ethnic clusters. PCA was applied to 1363 samples with 305,950 biallelic SNPs. The same PCA procedure was conducted on the validation cohort using a combined set of CAGI and 1,000 genome phase 1 data (209,029 biallelic SNPs across 1158 samples) and on the combined Kansas and 1,000 genome phase 1 data (224, 786 biallelic SNP across 1134 samples) to discriminate ethnic clusters.

### Variant calling and quality control

Next generation sequencing pipelines typically identify genomic locations at which any given sample *differs* from the human genome reference sequence on a case-by-case basis. After compiling the list of all variants identified in all cases and controls it was necessary to positively re-call the genotypic state (for the full set of all variants from all samples) in order to distinguish allelic genotypic status from missing data for each individual. The resultant genotypes were used for further analysis. Variants were excluded using vcftools if they deviated significantly from Hardy-Weinberg equilibrium status (p < 0.001) in the control group. Samples with a genotype missing call rate >95% were also excluded. VCF files containing genotypic information for all cases and controls were merged and annotated.

To detect association between genetic variant and disease status, a gene-based test (the sequence kernel association optimal unified test^26^, SKAT-O) was performed using the EPACTS software package^49^ in the discovery cohort. SKAT-O test was further conducted on the replication cohort to validate significant results from the discovery cohort.

### Burden of mutation testing in the discovery cohort

SKAT-O statistical test was applied to further investigate the joint effect of rare and low frequency variants. Specifically, SKAT-O encompasses both a burden test and a SKAT test to offer a powerful means of conducting association analyses on combined rare and common variation as single variant tests are often underpowered due to the large sample size needed to detect a significant association.

To conduct the test, a group file with mutations of interests (synonymous, non-synonymous, splicing, frameshifts and non-frameshifts, stop gain and stop loss) was created for each of the 41 genes. SKAT-O was executed with the small sample adjustment, by using a MAF threshold of 0.05 to define rare variations within the sample size and using default weights^26^.

### Burden of mutation testing in the validation cohort

As the validation cohort comprises of whole-exome and whole-genome subjects, only variants falling within the consensus target region were considered. By limiting variants assessed to only those found in the genomic regions captured by both technologies, we limited the potential for bias when using data from two different capture technologies. Variant sites across the four genes requiring replication were used to generate a subset of the VCF file for each dataset. Ultimately, VCF files for all individuals were merged and annotated. SKAT-O testing was conducted using the same settings applied in the discovery cohort.

SKAT-O testing was further conducted using the same approach on the combined discovery and replication cohorts (n_cases_ = 169 and n_controls_ = 217).

## Additional Information

**How to cite this article:** Andreoletti, G. *et al*. Exome Analysis of Rare and Common Variants within the NOD Signaling Pathway. *Sci. Rep.* **7**, 46454; doi: 10.1038/srep46454 (2017).

**Publisher's note:** Springer Nature remains neutral with regard to jurisdictional claims in published maps and institutional affiliations.

## Supplementary Material

Supplementary Information

## Figures and Tables

**Figure 1:**
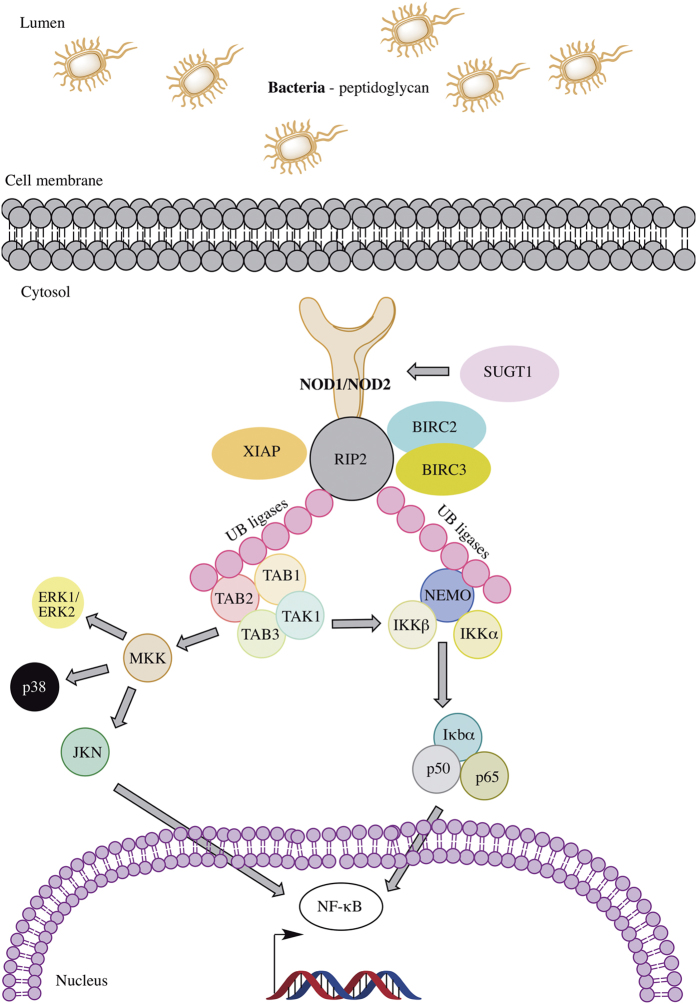
Proteins acting within the NOD signaling pathway. The recognistion of NOD1 and NOD2 of the bacterial peptidoglycan (PGN) promotes the formation of the multi-protein complex named inflammasome. The complex recruits the kinase receptor interacting protein 2 (RIP2), which is ubiquitinated by the ubiquitin ligases XIAP, BIRC2 and BIRC3 proteins. The polyubiquitinated RIP2 recruits the kinase TAK1 and TAK binding protein 1 (TAB1), TAB2 and TAB3 which leads to the activation of the MAPK kinases, p38 and c-Jun N-terminal kinase (JNK) through the activation of mitogen-activated protein kinase kinase (MKK). RIP2 polyubiquitinated also interacts with the IKK complex (IKKα, IKKβ and NEMO). The IKK complex mediates the phosphorylation of the IKKβ subunit of IKK by TAK1 and results in the phosphorylation and degradation of the NF-κB inhibitor (IκBα) which results in the cytoplasmic release and translocation of NF-κB dimers p65 and p50 in the nucleus to activate of the expression of the NF-κB proinflammatory genes.

**Table 1:** List of 31 NOD2 variants observed across the discovery cohort.

Bp position (hg19)	Vari-ant	Coding change	Protein change	Protein domain	SIFT pred-iction	Gerp	Max-Ent score	CA-DD	db-SNP	Frequ-ency in _1 KG_	Frequ-ency in NH-LBI ESP	Frequ-ency in Exac	HG-MD	Frequency in cases (n = 136)			Frequency in controls (n = 106)		
														Homo-zygous Refe-rence	Hetero-zygous	Homo-zygous Alter-native	Homo-zygous Refer-ence	Hetero-zygous	Homo-zygous Alter-native
5073-3392	sp	c.74–7T > A		Adjacent to CARD			1.83		rs1048-95421	0.0014	0.00-1861	0.00001	listed	1	0	0	0.99	0.01	0
5073-3423	ns	c.98C > A	p.A33D	CARD	T	1.13		1.40-2571		0.000008		0.00001	not listed	0.99	0.01	0	1	0	0
5073-3661	sn	c.336C > T	p.A112A	CARD						0.00002		0.00003	not listed	1	0	0	0.99	0.01	0
5073-3785	ns	c.460G > A	p.D154N	CARD	T	0.958		0.41-0941	rs146-054564		0.00-2093	0.00064	not listed	1	0	0	0.99	0.01	0
5073-3859	sn	c.534C > G	p.S178S	CARD					rs206-7085	0.26	0.40-9302	0.334	not listed	0.39	0.54	0.07	0.47	0.38	0.15
5074-1791	ns	c.566C > T	p.T189M	CARD	T	3.48		2.334-887	rs6175-5182	0.0014	0.00-4419	0.00259	listed	0.99	0.01	0	1	0	0
5074-1800	ns	c.575C > T	p.A192V	CARD	D	0.916		0.88-5309	rs1490-71116	0.00004		0.00005	not listed	1	0	0	0.99	0.01	0
5074-1858	sn	c.633C > T	p.A211A	CARD					rs574-3269	0.0009	0.00-1744	0.0008	not listed	1	0	0	0.99	0.01	0
5074-4624	ns	c.802C > T	p.P268S	NBD	T	−9.98		−0.27-189	rs206-6842	0.12	0.26-907	0.184	not listed	0.43	0.47	0.1	0.56	0.35	0.09
5074-4688	ns	c.866A > G	p.N289S	NBD	D	4.56	.	0.44-4188	rs574-3271	0.01	0.00-6279	0.00425	listed	0.99	0.01	0	0.98	0.02	0
5074-4850	ns	c.1028T > C	p.L343P	NBD	D	5.4		0.51-7926			0.00-0116	0.00001	not listed	1	0	0	0.99	0.01	0
5074-5114	ns	c.1292C > T	p.S431L	NBD	D	3.64		0.85-1472	rs1048-95431	0.0005	0.00-1395	0.00082	listed	0.99	0.01	0	1	0	0
5074-5199	sn	c.1377C > T	p.R459R	NBD					rs206-6843	0.13	0.27-0993	0.185	not listed	0.42	0.48	0.1	0.5	0.4	0.1
5074-5316	sn	c.1494A > G	p.E498E	NBD									not listed	1	0	0	0.99	0.01	0
5074-5511	sn	c.1689C > T	p.Y563Y	NBD					rs1116-08429	0.0005		0.00007	not listed	0.99	0.01	0	1	0	0
5074-5583	sn	c.1761T > G	p.R587R	NBD					rs186-|1759	0.25	0.40-2558	0.328	not listed	0.4	0.54	0.07	0.47	0.39	0.14
5074-5655	sn	c.1833C > T	p.A611A	NBD					rs617-36932	0.0046	0.01-0698	0.00983	not listed	0.99	0.01	0	1	0	0
5074-5751	sn	c.1929C > T	p.L643L	NBD						0.000008		0.00001	not listed	1	0	0	0.99	0.01	0
5074-5926	ns	c.2104C > T	p.R702W	NBD	D	2.42		1.73-6582	rs206-|6844	0.02	0.043-488	0.023	listed	0.88	0.1	0.01	0.87	0.13	0
5074-5929	ns	c.2107C > T	p.R703C	NBD	D	2.89		1.78-8325	rs574-3277	0.0023	0.00-6977	0.00002	listed	0.98	0.02	0	1	0	0
5074-5960	ns	c.2138G > A	p.R713H	NBD	T	4.13		2.22-5724	rs1048-95483		0.00-0233	0.00034	listed	0.99	0.01	0	1	0	0
5074-6086	ns	c.2264C > T	p.A755V	NBD	D	5.12		1.22-5314	rs6174-7625	0.0005	0.00-4651	0.00231	listed	0.99	0.01	0	1	0	0
5074-6199	ns	c.2377G > A	p.V793M	NBD	D	3.51		1.54-4959	rs1048-95444	0.0005	0.00-1628	0.00105	listed	0.99	0.01	0	0.98	0.02	0
5074-6228	sn	c.2406G > T	p.V802V	NBD				1.92-838	rs1048-95495		0.00-186	0.00196	not listed	0.99	0.01	0	0.99	0.01	0
5074-6291	sp	c.2462 + 7G > T		LRR			0.83		rs202-111813	0.0005	0.00-0581	0.00016	not listed	0.99	0.01	0	1	0	0
5075-0842	ns	c.2587A > G	p.M863V	LRR	T	−9.48			rs1048-95447		0.00186	0.0012	listed	0.99	0.01	0	1	0	0
5075-6540	ns	c.2722G > C	p.G908R	LRR	D	5.56			rs206-6845	0.01	0.01-4535	0.00992	listed	0.96	0.04	0	0.96	0.04	0
5075-6571	ns	c.2753C > A	p.A918D	LRR	D	5.56			rs1048-95452	0.0009	0.00-0814	0.0004	listed	1	0	0	0.99	0.01	0
5075-7276	ns	c.2863G > A	p.V955I	LRR	T	−9.14			rs574-3291	0.05	0.096-047	0.061	listed	0.83	0.17	0	0.81	0.19	0
5075-9405	ns	c.2888A > G	p.E963G	LRR	T	5.29							not listed	0.99	0.01	0	1	0	0
5076-3778	fr	c.3019-dupC	p.L1007fs	LRR					rs206-6847	0.006			listed	0.9	0.09	0.01	0.99	0.01	0

ns, non-synonymous; sn, synonymous; fi, Wframeshift insertion, fd, frameshift deletion; sp, splicing; nfi, non-frameshift insertion; nfd, non-frameshift deletion, sp, splicing. CARD, caspase recruitment domain; NBD, Nucleotide-binding oligomerization domain; LRR, leucine-rich domain. B, benign; C, Conservative; D, deleterious; MC, moderately Conservative; MR, moderately Radical; NR, not reported; P, possibly damaging; R, radical; T, tolerated.

**Table 2:** Joint variant test (SKAT-O) result for the 41 genes within the NOD signaling pathway in which variations was found across the entire discovery cohort.

Gene	Chromosome	bp position (hg19)	Total number of samples (136 cases; 106 controls)	Fraction of individuals who carry rare variants under the MAF thresholds (MAF < 0.05)*	Number of all variants defined in the group file	Number of variant defined as rare (MAF < 0.05)*	P-value unadjusted
BIRC2	11	102220918–102248410	242	0.07851	6	6	0.004
NFKB1	4	103488139–103537672	242	0.11983	10	9	0.005
NOD2	16	50733392–50763778	242	0.21488	31	25	0.029
SUGT1	13	53231709–53261936	242	0.33058	6	5	0.047
MAPK11	22	50703796–50706381	242	0.07024	7	5	0.061
CARD6	5	40841561–40853404	242	0.0909	10	8	0.074
BIRC3	11	102195774–102201850	242	0.05371	6	6	0.075
TAB2	6	149699333–149730846	242	0.07024	6	6	0.117
IKBKB	8	42128942–42188489	242	0.06198	6	6	0.249
ERBB2IP	5	65307924–65372200	242	0.17355	13	9	0.292
CASP8	2	202122956–202149864	242	0.0909	8	6	0.319
MAPK12	22	50691870–50699668	242	0.19835	16	13	0.35
TAB3	X	30849697–30877801	242	0.02066	5	4	0.362
CHUK	10	101964267–101980355	242	0.02892	6	4	0.38
TNFAIP3	6	138196066–138202378	242	0.06198	7	7	0.653
NOD1	7	30487954–30496518	242	0.08264	17	13	0.657
TRIP6	7	100465128–100469223	242	0.07438	11	9	0.783
TAB1	22	39795831–39832516	242	0.02479	7	6	0.795
MAPK13	6	36098410–36107131	242	0.02892	9	8	0.799
RIPK2	8	90770315–90802611	242	0.07438	5	4	0.803
CARD9	9	139258615–139266519	242	0.16942	12	10	0.866

Only genes in which at least five variants were entered into the model are shown.

*These variants received different weights in the SKAT-O joint test. Genes are ordered by p-value.

**Table 3:** SKAT-O test result for the four significant genes within the NOD signaling pathway in which variations was found across the replication cohort only and across the combined discovery and replication cohort.

Gene	Chromosome	bp position (hg19)	Dataset	Total number of samples	Fraction of individuals who carry rare variants under the MAF thresholds (MAF < 0.05)*	Number of all variants defined in the group file	Number of variant defined as rare (MAF < 0.05)*	P-value unadjusted
BIRC2	11	102219940–102249151	Replication cohort (33 cases; 111 controls)	144	0.11806	3	2	0.041
			Combined replication and validation cohort (169 cases; 217 controls)	386	0.04663	1	1	0. 030
NOD2	16	50733859–50763778	Replication cohort (33 cases; 111 controls)	144	0.11111	8	3	0.045
			Combined replication and validation cohort (169 cases; 217 controls)	386	0.041451	4	2	0.011
NFKB1	4	103505961–103514658	Replication cohort (33 cases; 111 controls)	144	0.02777	2	1	0.223
			Combined replication and validation cohort (169 cases; 217 controls)	386	0.05699	2	1	0.017

*These variants received different weights in the SKAT-O joint test. Genes are ordered by p-value.

**Table 4:** Patient demographics for 146 pediatric IBD patients that underwent whole-exome sequencing.

	CD	UC	IBDU
n	90	32	24
Male (%)	57 (63.3)	18 (56.25)	8 (33.3)
Mean age in years (range)	11.25 (2–17)	9.97 (1–15)	11.17 (2–16)

CD, Crohn's disease; UC, ulcerative colitis; IBDU, inflammatory bowel disease unclassified.
