# Supplementary material for: Exome Analysis of Rare and Common Variants within the NOD Signaling Pathway
Source: Sci Rep. 2017 Apr 19;7:46454. doi: 10.1038/srep46454 (PMC5396125; doi:10.1038/srep46454)
Supplement: Supplementary Information [file srep46454-s1.doc]

**Supplementary information**

# Exome Analysis of Rare and Common Variants within the NOD Signaling Pathway

Gaia Andreoletti, Valentina Shakhnovich, Kathy Christenson, Tracy Coelho, Rachel Haggarty, Nadeem A Afzal, Akshay Batra, Britt-Sabina Petersen, Matthew Mort, R Mark Beattie, Sarah Ennis

Supplementary

Figure 1. Principle component analysis (PCA) across five ethnic groups from 1000 genome project and the discovery cohort (146 pediatric IBD cases and 126 non-IBD controls).

**
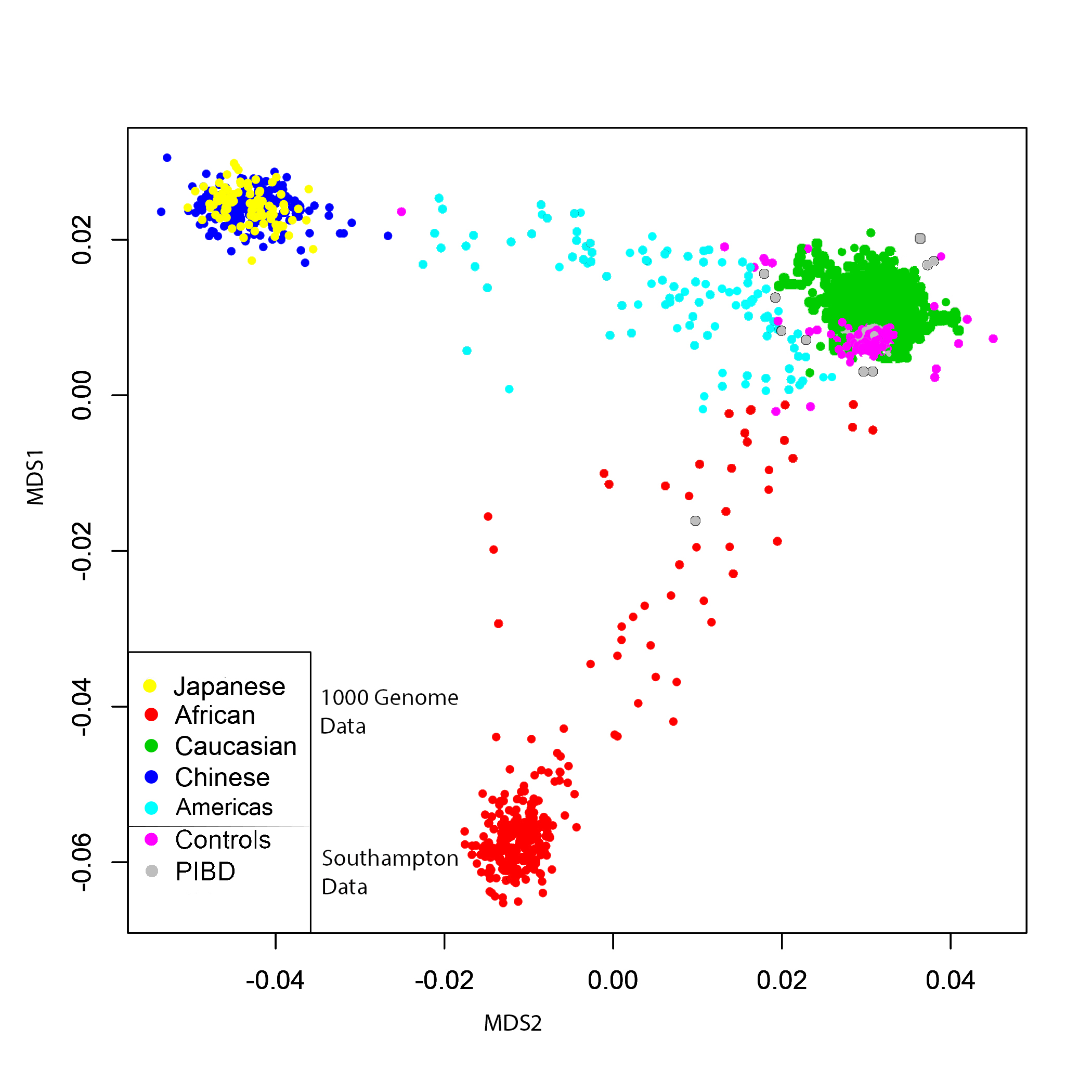
**

The five ethnic groups from 1000 Genome are colored as indicated. The Southampton IBD cohort and controls are in pink and light blue respectively. Southampton IBD samples excluded from the SKAT-O test because of ethnic status are represented with a black outline.

Figure 2. Principle component analysis (PCA) across five ethnic groups from 1000 genome project and the validation cohort (66 whole-exome data of the CAGI dataset).


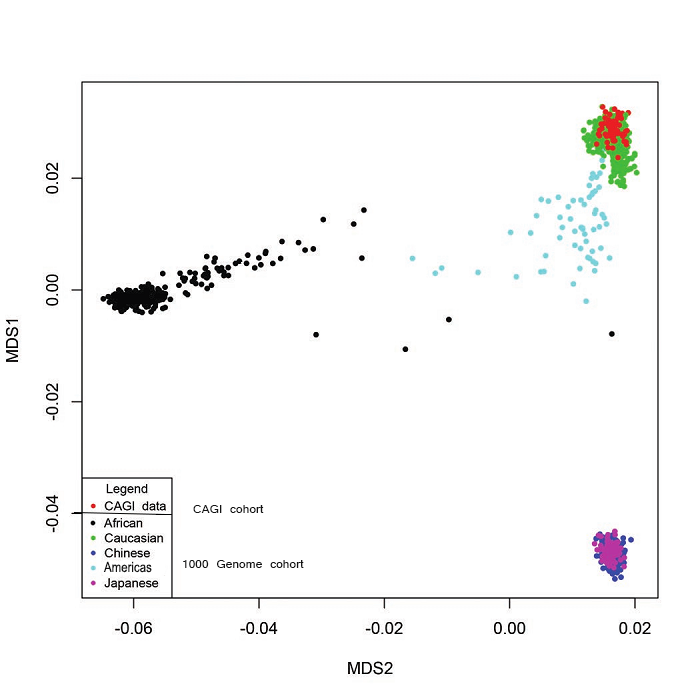


The five ethnic groups from 1000 Genome are colored as indicated. The CAGI cohort is in red.

Figure 3. Principle component analysis (PCA) across ethnic groups from 1000 genome project and the Kansas subgroup of the validation cohort (43 whole-exome data).


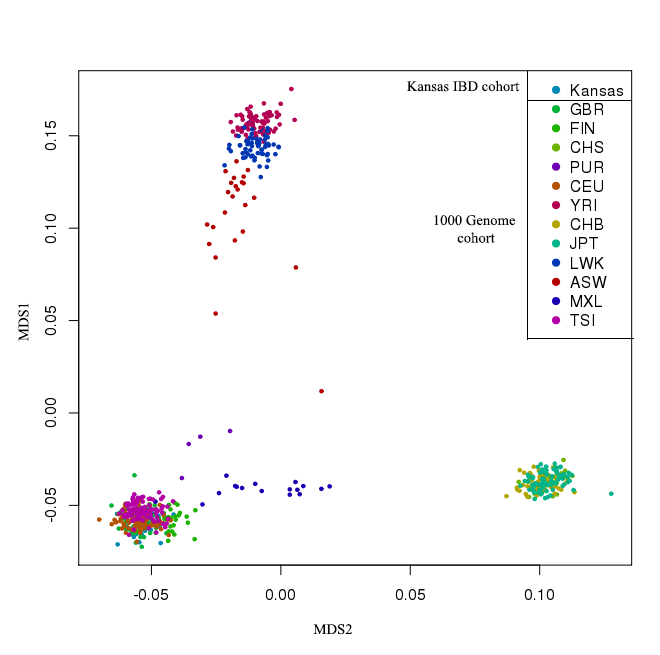


The ethnic groups from 1000 Genome are colored as indicated. The CAGI cohort is in blue.

Table S1. Percentage of gene coverage for each of the 40 genes involved in the NOD2 pathway according to the Agilent SureSelect V4 and Agilent SureSelect V5 all Human exome capture kits.

| **Gene** | **Protein** | **Agilent v4 % gene coverage** | **Agilent V5 % gene coverage** |
| --- | --- | --- | --- |
| *BIRC2* | BIRC2 (cIAP1) | 67.61 | 86.71 |
| *BIRC3* | BIRC3 (cIAP2) | 38.88 | 98.45 |
| *CARD6* | CARD6 | 82.43 | 99.85 |
| *CARD9* | CARD9 | 93.58 | 93.58 |
| *CASP8* | CASP8 | 82.56 | 84.88 |
| *CCL2** | CCL2 | 75.64 | 100.00 |
| *CCL5* | CCL5 | 45.00 | 80.39 |
| *CHUK* | IKKα | 72.19 | 100.00 |
| *CXCL1** | CXCL1 | 40.58 | 97.04 |
| *CXCL2** | CXCL2 | 38.90 | 100.00 |
| *ERBB2IP* | LAP2 | 64.45 | 95.90 |
| *IKBKB* | IKKβ | 76.52 | 88.15 |
| *IKBKG* | NEMO | 15.16 | 77.39 |
| *IL6* | IL6 | 83.11 | 100.00 |
| *IL8** | IL8 | 43.64 | 100.00 |
| *MAP3K7* | M3K7 | 43.22 | 98.78 |
| *MAPK1** | MK01 | 26.15 | 26.15 |
| *MAPK10* | MK10 | 30.51 | 86.73 |
| *MAPK11* | MK11 | 57.24 | 100.00 |
| *MAPK12* | MK12 | 85.12 | 100.00 |
| *MAPK13* | MK13 | 23.00 | 30.28 |
| *MAPK14* | MK14 | 39.65 | 100.00 |
| *MAPK3* | MK03 | 73.08 | 73.08 |
| *MAPK8* | MK08 | 100.00 | 100.00 |
| *MAPK9* | MK09 | 25.89 | 97.46 |
| *NFKB1** | NFKB1/p50 | 75.48 | 88.84 |
| *NFKBIA* | IKBα | 85.19 | 100.00 |
| *NFKBIB* | IKBB | 68.85 | 71.69 |
| *NOD1* | NOD1 | 71.09 | 91.06 |
| *NOD2** | NOD2 | 77.79 | 100.00 |
| *RELA** | TF65 | 61.86 | 100.00 |
| *RIPK2** | RIPs2 | 73.81 | 100.00 |
| *SUGT1* | SUGT1 | 73.74 | 98.32 |
| *TAB1** | TAB1 | 54.17 | 95.55 |
| *TAB2* | TAB2 | 53.90 | 92.45 |
| *TAB3* | TAB3 | 43.80 | 91.56 |
| *TNF* | TNFα | 9.72 | 12.84 |
| *TNFAIP3** | TNAP3 | 47.21 | 81.53 |
| *TRAF6* | TRAF6 | 22.58 | 55.42 |
| *TRIP6* | TRIP6 | 100.00 | 100.00 |
| *XIAP* | XIAP | 15.50 | 92.70 |

Genes with asterisks indicate genes previously identified by GWAS.

Table S2. List of 250 variants found within the 40 genes of the NOD2 pathway in which variations was found across the entire cohort of 136 cases and 106 controls.

| Gene | Chr | Bp position (hg19) | Variant. | Coding change | Protein change | SIFT | Gerp | MaxEnt score | CADD | dbSNP | Frequency in 1KG | Frequency in NHLBI ESP | Frequency in Exac | HGMD | Frequency in cases (n=136) | | | Frequency in controls (n=106) | | |
| --- | --- | --- | --- | --- | --- | --- | --- | --- | --- | --- | --- | --- | --- | --- | --- | --- | --- | --- | --- | --- |
|  |  |  |  |  |  |  |  |  |  |  |  |  |  |  | Homozygous reference | Heterozygous | Homozygous alternative | Homozygous reference | Heterozygous | Homozygous alternative |
| BIRC2 | 11 | 102220918 | fr | c.335_339del | p.112_113del | . | . | . | . | . | . | . | . | not listed | 99.26 | 0.74 | 0 | 100 | 0 | 0 |
| BIRC2 | 11 | 102221045 | ns | c.460T>G | p.S154A | T | 2.14 | . | 0.553713 | . | . | . | 0.00006 | not listed | 100 | 0 | 0 | 98.11 | 1.89 | 0 |
| BIRC2 | 11 | 102221633 | sn | c.954T>C | p.S318S | . | . | . | . | rs182906329 | 0.0014 | 0.001163 | 0.00133 | not listed | 99.26 | 0.74 | 0 | 98.11 | 1.89 | 0 |
| BIRC2 | 11 | 102248377 | ns | c.1517C>T | p.A506V | D | 4.65 | . | 3.506238 | rs34510872 | 0.03 | 0.052117 | 0.04900 | not listed | 92.65 | 7.35 | 0 | 100 | 0 | 0 |
| BIRC2 | 11 | 102248406 | ns | c.1546A>G | p.K516E | D | 5.61 | . | 5.497399 | rs61754131 | 0.0005 | 0.001745 | 0.00128 | not listed | 98.53 | 1.47 | 0 | 100 | 0 | 0 |
| BIRC2 | 11 | 102248410 | ns | c.1550G>A | p.G517E | D | 5.61 | . | 4.831211 | . | . | . | . | not listed | 99.26 | 0.74 | 0 | 100 | 0 | 0 |
| BIRC3 | 11 | 102195882 | sn | c.642T>C | p.N214N | . | . | . | . | . | . | . | 0.00005 | not listed | 99.26 | 0.74 | 0 | 100 | 0 | 0 |
| BIRC3 | 11 | 102195897 | sn | c.657T>C | p.D219D | . | . | . | . | . | . | . | . | not listed | 99.26 | 0.74 | 0 | 98.11 | 1.89 | 0 |
| BIRC3 | 11 | 102196019 | ns | c.779A>G | p.K260R | T | -3.66 | . | -0.04809 | rs2276113 | 0.02 | 0.000814 | 0.01900 | not listed | 100 | 0 | 0 | 99.06 | 0.94 | 0 |
| BIRC3 | 11 | 102201804 | ns | c.1156G>A | p.V386M | D | 2.37 | . | 0.619961 | rs12222256 | 0.0023 | . | 0.00047 | not listed | 100 | 0 | 0 | 99.06 | 0.94 | 0 |
| BIRC3 | 11 | 102201848 | sn | c.1200G>A | p.Q400Q | . | . | . | . | rs17878663 | 0.07 | 0.001163 | 0.03400 | not listed | 100 | 0 | 0 | 99.06 | 0.94 | 0 |
| BIRC3 | 11 | 102201850 | ns | c.1202G>A | p.R401K | T | 4.08 | . | 2.353145 | rs17881197 | 0.0041 | 0.008258 | 0.00586 | not listed | 98.53 | 1.47 | 0 | 95.28 | 4.72 | 0 |
| CARD6 | 5 | 40841561 | ns | c.77C>G | p.P26R | D | 3.66 | . | 1.550447 | . | . | . | . | not listed | 99.26 | 0.74 | 0 | 100 | 0 | 0 |
| CARD6 | 5 | 40841571 | fr | c.88_89del | p.30_30del | . | . | . | . | rs141244584 | 0.01 | . | 0.02000 | not listed | 95.59 | 4.41 | 0 | 97.17 | 2.83 | 0 |
| CARD6 | 5 | 40841741 | ns | c.257C>T | p.S86L | D | 3.74 | . | 1.737134 | rs10512747 | 0.06 | 0.120465 | 0.09100 | not listed | 76.47 | 21.32 | 2.21 | 77.36 | 19.81 | 2.83 |
| CARD6 | 5 | 40843493 | ns | c.523A>T | p.T175S | D | -2.97 | . | -1.70298 | rs61748215 | 0.0037 | 0.01 | 0.01200 | not listed | 98.53 | 1.47 | 0 | 99.06 | 0.94 | 0 |
| CARD6 | 5 | 40843550 | ns | c.580A>G | p.I194V | T | 3.51 | . | 1.02021 | rs61757654 | 0.01 | 0.013023 | 0.00872 | not listed | 97.79 | 2.21 | 0 | 100 | 0 | 0 |
| CARD6 | 5 | 40843735 | ns | c.765C>A | p.F255L | D | 1.41 | . | 2.570366 | rs35188876 | 0.01 | 0.018953 | 0.01400 | not listed | 97.06 | 2.94 | 0 | 99.06 | 0.94 | 0 |
| CARD6 | 5 | 40852317 | ns | c.883A>G | p.M295V | D | -0.882 | . | 1.770901 | rs61748217 | 0.0009 | 0.00186 | 0.00158 | not listed | 97.79 | 2.21 | 0 | 100 | 0 | 0 |
| CARD6 | 5 | 40852902 | ns | c.1468G>A | p.D490N | T | 4.85 | . | . | . | . | . | . | not listed | 100 | 0 | 0 | 99.06 | 0.94 | 0 |
| CARD6 | 5 | 40853048 | sn | c.1614C>T | p.S538S | . | . | . | . | rs16870407 | 0.15 | 0.092791 | 0.13800 | not listed | 83.82 | 15.44 | 0.74 | 80.19 | 18.87 | 0.94 |
| CARD6 | 5 | 40853404 | ns | c.1970T>C | p.V657A | D | 4.18 | . | 4.484833 | . | . | . | . | not listed | 99.26 | 0.74 | 0 | 100 | 0 | 0 |
| CARD9 | 9 | 139258779 | ns | c.1586A>C | p.D529A | T | 2.88 | . | 2.038243 | . | . | . | . | not listed | 99.26 | 0.74 | 0 | 100 | 0 | 0 |
| CARD9 | 9 | 139258814 | ns | c.1551G>C | p.Q517H | T | 1.57 | . | 0.959136 | . | . | . | 0.00021 | not listed | 100 | 0 | 0 | 99.06 | 0.94 | 0 |
| CARD9 | 9 | 139259644 | sn | c.1383G>A | p.P461P | . | . | . | . | rs138344913 | 0.0018 | 0.003373 | 0.00002 | not listed | 100 | 0 | 0 | 99.06 | 0.94 | 0 |
| CARD9 | 9 | 139262205 | ns | c.1153G>C | p.V385L | T | 1.37 | . | 0.261356 | rs3124993 | 0.01 | 0.021567 | 0.01600 | not listed | 94.85 | 5.15 | 0 | 95.28 | 4.72 | 0 |
| CARD9 | 9 | 139264888 | ns | c.809A>T | p.E270V | T | 2.85 | . | 0.381144 | rs114895119 | 0.0032 | 0.004307 | 0.00350 | not listed | 100 | 0 | 0 | 99.06 | 0.94 | 0 |
| CARD9 | 9 | 139265088 | sn | c.693G>A | p.T231T | . | . | . | . | rs59902911 | 0.06 | 0.031177 | 0.03900 | not listed | 94.12 | 5.88 | 0 | 89.62 | 10.38 | 0 |
| CARD9 | 9 | 139265801 | sn | c.297G>A | p.P99P | . | . | . | . | rs115131813 | 0.03 | 0.017113 | 0.02500 | not listed | 95.59 | 4.41 | 0 | 97.17 | 2.83 | 0 |
| CARD9 | 9 | 139265810 | sn | c.288C>A | p.G96G | . | . | . | . | rs137986801 | . | 0.002096 | 0.00111 | not listed | 99.26 | 0.74 | 0 | 99.06 | 0.94 | 0 |
| CARD9 | 9 | 139265870 | sn | c.228C>T | p.Y76Y | . | . | . | . | rs11145769 | 0.06 | 0.031585 | 0.03600 | not listed | 94.12 | 5.88 | 0 | 91.51 | 8.49 | 0 |
| CARD9 | 9 | 139266405 | sn | c.126C>T | p.P42P | . | . | . | . | rs10781499 | 0.37 | 0.423372 | 0.40100 | not listed | 34.56 | 43.38 | 22.06 | 34.91 | 49.06 | 16.04 |
| CARD9 | 9 | 139266496 | ns | c.35G>A | p.S12N | T | -0.646 | . | 0.446262 | rs4077515 | 0.37 | 0.421512 | 0.39900 | listed | 34.56 | 43.38 | 22.06 | 37.74 | 46.23 | 16.04 |
| CARD9 | 9 | 139266519 | sn | c.12C>T | p.Y4Y | . | . | . | . | rs35051231 | 0.0005 | 0.001163 | 0.00054 | not listed | 99.26 | 0.74 | 0 | 100 | 0 | 0 |
| CASP8 | 2 | 202122956 | ns | c.2T>C | p.M1T | D | -0.239 | . | 0.151387 | rs3769824 | 0.03 | 0.043705 | 0.04500 | not listed | 94.85 | 5.15 | 0 | 88.68 | 11.32 | 0 |
| CASP8 | 2 | 202122995 | ns | c.41A>G | p.K14R | D | 2.94 | . | 0.164368 | rs3769823 | 0.65 | 0.705882 | 0.66700 | not listed | 16.18 | 38.24 | 45.59 | 11.32 | 43.4 | 45.28 |
| CASP8 | 2 | 202123108 | sp | c.151+3G>A | . | . | . | 0.85 | . | rs202238412 | . | 0.001798 | 0.00076 | not listed | 99.26 | 0.74 | 0 | 100 | 0 | 0 |
| CASP8 | 2 | 202136272 | sn | c.339C>T | p.S113S | . | . | . | . | rs17860422 | 0.0023 | 0.00407 | 0.00360 | not listed | 98.53 | 1.47 | 0 | 100 | 0 | 0 |
| CASP8 | 2 | 202137392 | ns | c.443A>G | p.K148R | T | 5.52 | . | 3.96662 | rs148697064 | . | 0.000698 | 0.00033 | not listed | 100 | 0 | 0 | 99.06 | 0.94 | 0 |
| CASP8 | 2 | 202149589 | ns | c.808G>C | p.D270H | T | -4.55 | . | -0.30191 | rs1045485 | 0.07 | 0.129186 | 0.09100 | listed | 77.94 | 21.32 | 0.74 | 83.96 | 16.04 | 0 |
| CASP8 | 2 | 202149696 | sn | c.915G>A | p.K305K | . | . | . | . | rs1045487 | 0.15 | 0.051047 | 0.10200 | not listed | 91.91 | 8.09 | 0 | 85.85 | 14.15 | 0 |
| CASP8 | 2 | 202149737 | ns | c.956A>G | p.Y319C | D | -1.54 | . | 0.964215 | . | . | . | 0.00004 | not listed | 100 | 0 | 0 | 99.06 | 0.94 | 0 |
| CCL2 | 17 | 32583269 | sn | c.105T>C | p.C35C | . | . | . | . | rs4586 | 0.54 | 0.36093 | 0.44200 | not listed | 49.26 | 43.38 | 7.35 | 44.34 | 39.62 | 16.04 |
| CHUK | 10 | 101964267 | sn | c.1503G>A | p.G501G | . | . | . | . | rs2862988 | 0.0018 | 0.00593 | 0.00666 | not listed | 99.26 | 0.74 | 0 | 97.17 | 2.83 | 0 |
| CHUK | 10 | 101964312 | sn | c.1458C>T | p.S486S | . | . | . | . | rs17880383 | 0.04 | 0.061395 | 0.04000 | not listed | 87.5 | 11.03 | 1.47 | 94.34 | 5.66 | 0 |
| CHUK | 10 | 101964847 | sn | c.1341A>G | p.G447G | . | . | . | . | rs34458357 | 0.0014 | 0.005465 | 0.00291 | not listed | 99.26 | 0.74 | 0 | 100 | 0 | 0 |
| CHUK | 10 | 101964950 | ns | c.1238A>C | p.D413A | T | 5.01 | . | 4.795139 | . | . | . | . | not listed | 99.26 | 0.74 | 0 | 100 | 0 | 0 |
| CHUK | 10 | 101977883 | ns | c.802G>A | p.V268I | T | 4.69 | . | 2.550085 | rs2230804 | 0.56 | 0.501047 | 0.47900 | not listed | 21.32 | 52.21 | 26.47 | 28.3 | 50 | 21.7 |
| CHUK | 10 | 101980355 | ns | c.464T>C | p.V155A | T | 4.16 | . | 1.072682 | rs2230803 | 0.02 | 0.001163 | 0.01300 | not listed | 100 | 0 | 0 | 99.06 | 0.94 | 0 |
| CXCL1 | 4 | 74735244 | sn | c.57A>G | p.A19A | . | . | . | . | rs2071425 | 0.34 | 0.186713 | 0.26500 | not listed | 54.41 | 40.44 | 5.15 | 72.64 | 26.42 | 0.94 |
| CXCL1 | 4 | 74736235 | sp | c.309-3C>T | . | . | . | 1.03 | . | rs1814092 | 0.05 | 0.001628 | 0.02000 | not listed | 98.53 | 1.47 | 0 | 100 | 0 | 0 |
| CXCL2 | 4 | 74964625 | ns | c.115A>G | p.T39A | T | -0.319 | . | 0.437763 | rs142264518 | 0.0046 | 0.006977 | 0.00528 | not listed | 98.53 | 1.47 | 0 | 99.06 | 0.94 | 0 |
| CXCL2 | 4 | 74964830 | ns | c.8G>A | p.R3H | T | -5.75 | . | -0.01658 | rs186397980 | 0.02 | 0.015973 | 0.00546 | not listed | 97.79 | 1.47 | 0.74 | 97.17 | 2.83 | 0 |
| ERBB2IP | 5 | 65307924 | ns | c.355A>G | p.I119V | T | -2.81 | . | 0.260566 | rs61758158 | 0.01 | 0.01143 | 0.00962 | not listed | 95.59 | 3.68 | 0.74 | 97.17 | 2.83 | 0 |
| ERBB2IP | 5 | 65317181 | sn | c.565C>T | p.L189L | . | . | . | . | rs706679 | 0.67 | 0.856478 | 0.77000 | not listed | 11.76 | 12.5 | 75.74 | 10.38 | 31.13 | 58.49 |
| ERBB2IP | 5 | 65317206 | ns | c.590C>T | p.T197M | D | 4.58 | . | 5.955216 | rs146136641 | 0.0005 | 0.001512 | 0.00069 | listed | 99.26 | 0.74 | 0 | 100 | 0 | 0 |
| ERBB2IP | 5 | 65321311 | ns | c.821C>T | p.S274L | T | 2.41 | . | 1.987188 | rs3213837 | 0.1 | 0.156315 | 0.15400 | not listed | 77.94 | 19.12 | 2.94 | 67.92 | 31.13 | 0.94 |
| ERBB2IP | 5 | 65349300 | sn | c.2142A>G | p.E714E | . | . | . | . | . | . | . | . | not listed | 99.26 | 0.74 | 0 | 100 | 0 | 0 |
| ERBB2IP | 5 | 65349887 | ns | c.2729A>G | p.K910R | D | 4.46 | . | . | rs34521887 | 0.01 | 0.000116 | 0.00315 | not listed | 99.26 | 0.74 | 0 | 100 | 0 | 0 |
| ERBB2IP | 5 | 65350044 | sn | c.2886A>G | p.Q962Q | . | . | . | . | rs35278406 | 0.02 | 0.039893 | 0.02800 | not listed | 90.44 | 9.56 | 0 | 91.51 | 8.49 | 0 |
| ERBB2IP | 5 | 65350279 | ns | c.3121C>T | p.H1041Y | D | 4.05 | . | 1.755784 | rs142496054 | 0.01 | 0.008023 | 0.00705 | not listed | 99.26 | 0.74 | 0 | 96.23 | 2.83 | 0.94 |
| ERBB2IP | 5 | 65350374 | sn | c.3216A>G | p.R1072R | . | . | . | . | rs36303 | 0.23 | 0.138488 | 0.19200 | not listed | 80.15 | 17.65 | 2.21 | 65.09 | 31.13 | 3.77 |
| ERBB2IP | 5 | 65350481 | ns | c.3323C>T | p.S1108L | D | 5.32 | . | 4.6549 | rs3805466 | 0.1 | 0.047674 | 0.08500 | not listed | 91.18 | 6.62 | 2.21 | 76.42 | 22.64 | 0.94 |
| ERBB2IP | 5 | 65350527 | sn | c.3369T>G | p.L1123L | . | . | . | 0.161838 | . | . | 0.000581 | 0.00034 | not listed | 99.26 | 0.74 | 0 | 100 | 0 | 0 |
| ERBB2IP | 5 | 65370927 | ns | c.3697C>G | p.Q1233E | D | 5.34 | . | 2.367375 | rs201285970 | . | . | 0.00003 | not listed | 99.26 | 0.74 | 0 | 100 | 0 | 0 |
| ERBB2IP | 5 | 65372200 | sn | c.3885C>T | p.V1295V | . | . | . | . | . | . | . | 0.00002 | not listed | 100 | 0 | 0 | 98.11 | 1.89 | 0 |
| IKBKB | 8 | 42128942 | sn | c.54C>T | p.F18F | . | . | . | . | rs12545246 | 0.03 | . | 0.01100 | not listed | 97.06 | 2.94 | 0 | 99.06 | 0.94 | 0 |
| IKBKB | 8 | 42128970 | ns | c.82C>T | p.P28S | . | . | . | 0.730313 | . | . | . | 0.00012 | not listed | 100 | 0 | 0 | 99.06 | 0.94 | 0 |
| IKBKB | 8 | 42163863 | sn | c.303A>G | p.L101L | . | . | . | . | rs17875704 | 0.0009 | . | 0.00037 | not listed | 99.26 | 0.74 | 0 | 100 | 0 | 0 |
| IKBKB | 8 | 42174380 | sn | c.1077G>A | p.L359L | . | . | . | . | rs56230731 | 0.01 | 0.014884 | 0.00956 | not listed | 97.06 | 2.94 | 0 | 100 | 0 | 0 |
| IKBKB | 8 | 42178343 | ns | c.1663G>A | p.G555R | T | 4.96 | . | 3.028211 | rs149701177 | . | 0.000465 | 0.00002 | not listed | 100 | 0 | 0 | 98.11 | 1.89 | 0 |
| IKBKB | 8 | 42179427 | sn | c.1696A>C | p.R566R | . | . | . | . | rs151057347 | 0.0023 | 0.003837 | 0.00159 | not listed | 99.26 | 0.74 | 0 | 99.06 | 0.94 | 0 |
| IKBKG | X | 153780386 | ns | c.169G>A | p.E57K | D | 5.17 | . | 2.125638 | rs148695964 | . | 0.001784 | 0.00113 | listed | 99.26 | 0.74 | 0 | 100 | 0 | 0 |
| IL6 | 7 | 22771038 | ns | c.485A>T | p.D162V | T | 0.258 | . | -0.30563 | rs2069860 | 0.0018 | 0.00814 | 0.00612 | not listed | 97.79 | 2.21 | 0 | 98.11 | 1.89 | 0 |
| IL6 | 7 | 22771039 | ns | c.486T>A | p.D162E | T | -2.22 | . | 0.028822 | rs13306435 | 0.03 | 0.008837 | 0.02500 | not listed | 99.26 | 0.74 | 0 | 98.11 | 1.89 | 0 |
| IL6 | 7 | 22771156 | sn | c.603C>T | p.F201F | . | . | . | . | rs2069849 | 0.06 | 0.023605 | 0.04400 | not listed | 96.32 | 3.68 | 0 | 92.45 | 6.6 | 0.94 |
| IL8 | 4 | 74606393 | sn | c.18C>T | p.A6A | . | . | . | . | rs1803205 | . | 0.001977 | 0.00129 | not listed | 100 | 0 | 0 | 98.11 | 1.89 | 0 |
| IL8 | 4 | 74607328 | ns | c.134A>G | p.H45R | D | 3.7 | . | . | rs139503118 | . | . | 0.00024 | not listed | 99.26 | 0.74 | 0 | 100 | 0 | 0 |
| MAP3K7 | 6 | 91256978 | sn | c.1209A>G | p.T403T | . | . | . | . | . | . | 0.000116 | 0.00003 | not listed | 100 | 0 | 0 | 100 | 0 | 0 |
| MAP3K7 | 6 | 91266350 | sp | c.483-7T>A | . | . | . | 2.01 | . | rs45625637 | 0.06 | . | 0.08900 | not listed | 100 | 0 | 0 | 100 | 0 | 0 |
| MAPK1 | 22 | 22123519 | ns | c.1057A>G | p.R353G | T | 4.04 | . | 2.942178 | . | . | . | . | not listed | 100 | 0 | 0 | 99.06 | 0.94 | 0 |
| MAPK1 | 22 | 22142659 | sg | c.743C>A | p.S248X | . | 5.57 | . | 2.569548 | . | . | . | . | not listed | 100 | 0 | 0 | 99.06 | 0.94 | 0 |
| MAPK1 | 22 | 22160301 | sn | c.330A>G | p.T110T | . | . | . | . | rs150378600 | 0.0009 | 0.001163 | 0.00192 | not listed | 99.26 | 0.74 | 0 | 100 | 0 | 0 |
| MAPK1 | 22 | 22162126 | sn | c.129T>C | p.Y43Y | . | . | . | . | rs3729910 | 0.04 | 0.060698 | 0.04500 | not listed | 88.24 | 11.03 | 0.74 | 85.85 | 14.15 | 0 |
| MAPK1 | 22 | 22221708 | nonfi | c.2_3insGGC | p.M1delinsMA | . | . | . | . | . | . | . | . | not listed | 99.26 | 0.74 | 0 | 100 | 0 | 0 |
| MAPK10 | 4 | 86952589 | sp | c.1111-5GA | . | . | . | 1.08 | . | rs200643314 | . | . | . | not listed | 99.26 | 0.74 | 0 | 100 | 0 | 0 |
| MAPK10 | 4 | 86952590 | sp | c.1111-6CT | . | . | . | 0.61 | . | rs13103861 | 0.14 | 0.179884 | 0.15700 | not listed | 64.71 | 33.09 | 2.21 | 66.98 | 30.19 | 2.83 |
| MAPK11 | 22 | 50703796 | sn | c.969T>C | p.Y323Y | . | . | . | . | rs139548825 | . | 0.000581 | 0.00021 | not listed | 99.26 | 0.74 | 0 | 100 | 0 | 0 |
| MAPK11 | 22 | 50704028 | ns | c.824G>A | p.R275H | T | -3.93 | . | 1.290414 | rs33932986 | 0.02 | 0.019419 | 0.01800 | not listed | 97.79 | 1.47 | 0.74 | 98.11 | 1.89 | 0 |
| MAPK11 | 22 | 50704661 | sn | c.756A>G | p.S252S | . | . | . | . | rs2076139 | 0.7 | 0.762116 | 0.71500 | not listed | 5.88 | 37.5 | 56.62 | 18.87 | 27.36 | 53.77 |
| MAPK11 | 22 | 50705466 | sn | c.507T>C | p.F169F | . | . | . | . | rs760748 | 0.97 | 0.994416 | 0.99700 | not listed | 0 | 2.21 | 97.79 | 5.66 | 0.94 | 93.4 |
| MAPK11 | 22 | 50705821 | sn | c.396C>T | p.Y132Y | . | . | . | . | rs2066762 | 0.03 | 0.002214 | 0.01100 | not listed | 99.26 | 0.74 | 0 | 100 | 0 | 0 |
| MAPK11 | 22 | 50705830 | sn | c.387C>T | p.F129F | . | . | . | . | rs140519122 | . | 0.002098 | 0.00176 | not listed | 99.26 | 0.74 | 0 | 100 | 0 | 0 |
| MAPK11 | 22 | 50706381 | sp | c.117-3C>T | . | . | . | 0.57 | . | rs36083586 | 0.12 | 0.112509 | 0.11700 | not listed | 72.06 | 25 | 2.94 | 84.91 | 13.21 | 1.89 |
| MAPK12 | 22 | 50691870 | ns | c.1064G>A | p.R355Q | T | 0.925 | . | . | rs138582408 | 0.0027 | 0.002749 | 0.00299 | not listed | 100 | 0 | 0 | 97.17 | 2.83 | 0 |
| MAPK12 | 22 | 50691914 | sp | c.1025-5GC | . | . | . | 1.14 | . | . | . | . | . | not listed | 97.79 | 2.21 | 0 | 97.17 | 2.83 | 0 |
| MAPK12 | 22 | 50691915 | sp | c.1025-6CT | . | . | . | 0.07 | . | . | . | . | 0.00001 | not listed | 100 | 0 | 0 | 98.11 | 1.89 | 0 |
| MAPK12 | 22 | 50693619 | sp | c.1024+7GT | . | . | . | 0.92 | . | . | . | . | 0.00007 | not listed | 99.26 | 0.74 | 0 | 100 | 0 | 0 |
| MAPK12 | 22 | 50693705 | sn | c.945C>T | p.H315H | . | . | . | . | rs45606035 | 0.0018 | 0.005349 | 0.00277 | not listed | 100 | 0 | 0 | 99.06 | 0.94 | 0 |
| MAPK12 | 22 | 50693889 | sn | c.843C>T | p.S281S | . | . | . | . | rs2066770 | 0.04 | 0.018721 | 0.02800 | not listed | 95.59 | 4.41 | 0 | 97.17 | 2.83 | 0 |
| MAPK12 | 22 | 50693919 | sn | c.813G>A | p.K271K | . | . | . | . | rs55861809 | 0.01 | . | 0.00206 | not listed | 100 | 0 | 0 | 99.06 | 0.94 | 0 |
| MAPK12 | 22 | 50693934 | sn | c.798C>T | p.P266P | . | . | . | . | rs62239359 | 0.0009 | 0.005116 | 0.00311 | not listed | 98.53 | 1.47 | 0 | 100 | 0 | 0 |
| MAPK12 | 22 | 50694084 | ns | c.731C>T | p.T244M | D | 4.2 | . | . | rs2066776 | . | 0.002209 | 0.00109 | not listed | 99.26 | 0.74 | 0 | 100 | 0 | 0 |
| MAPK12 | 22 | 50694297 | sn | c.633T>C | p.S211S | . | . | . | . | rs1129880 | 0.79 | 0.704281 | 0.77300 | not listed | 8.09 | 41.18 | 50.74 | 20.75 | 33.02 | 46.23 |
| MAPK12 | 22 | 50694542 | sn | c.591C>T | p.I197I | . | . | . | . | . | . | . | 0.00006 | not listed | 99.26 | 0.74 | 0 | 100 | 0 | 0 |
| MAPK12 | 22 | 50694578 | sn | c.555C>T | p.Y185Y | . | . | . | . | . | . | . | 0.00003 | not listed | 100 | 0 | 0 | 99.06 | 0.94 | 0 |
| MAPK12 | 22 | 50695370 | sn | c.450C>T | p.I150I | . | . | . | . | rs2072876 | 0.04 | 0.023166 | 0.03400 | not listed | 94.12 | 5.15 | 0.74 | 95.28 | 4.72 | 0 |
| MAPK12 | 22 | 50696678 | ns | c.308C>T | p.T103M | T | 2.11 | . | . | rs34422484 | 0.09 | 0.031047 | 0.06100 | not listed | 89.71 | 10.29 | 0 | 95.28 | 4.72 | 0 |
| MAPK12 | 22 | 50699668 | sn | c.183T>C | p.P61P | . | . | . | . | rs2272857 | 0.68 | 0.769446 | 0.71100 | not listed | 5.88 | 36.76 | 57.35 | 24.53 | 26.42 | 49.06 |
| MAPK13 | 6 | 36098410 | sn | c.51A>C | p.T17T | . | . | . | . | rs1059227 | 0.77 | 0.660191 | 0.68300 | not listed | 8.09 | 46.32 | 45.59 | 21.7 | 36.79 | 41.51 |
| MAPK13 | 6 | 36098434 | sg | c.75C>A | p.Y25X | NA | -0.985 | . | 2.114124 | rs151226715 | 0.0014 | 0.000698 | 0.00082 | not listed | 99.26 | 0.74 | 0 | 99.06 | 0.94 | 0 |
| MAPK13 | 6 | 36098481 | sp | c.119+3>GA | . | . | . | 2.78 | . | rs140374075 | 0.01 | 0.001865 | 0.00471 | not listed | 99.26 | 0.74 | 0 | 100 | 0 | 0 |
| MAPK13 | 6 | 36099050 | ns | c.122C>T | p.S41L | D | 2.66 | . | 2.103868 | rs55776345 | 0.01 | 0.012907 | 0.00946 | not listed | 99.26 | 0.74 | 0 | 97.17 | 2.83 | 0 |
| MAPK13 | 6 | 36100425 | ns | c.277C>T | p.P93S | T | 1.67 | . | 2.505966 | rs148256444 | . | . | 0.00005 | not listed | 99.26 | 0.74 | 0 | 100 | 0 | 0 |
| MAPK13 | 6 | 36104430 | sp | c.496-3>TC | . | . | . | 0.27 | . | rs55732669 | 0.02 | 0.000349 | 0.00398 | not listed | 99.26 | 0.74 | 0 | 98.11 | 1.89 | 0 |
| MAPK13 | 6 | 36104455 | ns | c.518G>A | p.R173Q | D | 4.53 | . | 1.817101 | . | . | . | 0.00007 | not listed | 99.26 | 0.74 | 0 | 100 | 0 | 0 |
| MAPK13 | 6 | 36104502 | sg | c.565C>T | p.R189X | NA | 4.53 | . | 2.531246 | rs148572287 | . | 0.000116 | 0.00004 | not listed | 99.26 | 0.74 | 0 | 100 | 0 | 0 |
| MAPK13 | 6 | 36107131 | ns | c.1079G>A | p.R360Q | T | 2.53 | . | 2.12748 | rs150915766 | . | 0.001744 | 0.00125 | not listed | 99.26 | 0.74 | 0 | 100 | 0 | 0 |
| MAPK14 | 6 | 36063793 | ns | c.712C>T | p.L238F | D | 4.75 | . | 2.393865 | rs139802452 | . | 0.000465 | 0.00037 | not listed | 98.53 | 1.47 | 0 | 100 | 0 | 0 |
| MAPK14 | 6 | 36068038 | sn | c.756C>T | p.S252S | . | . | . | . | . | . | . | . | not listed | 99.26 | 0.74 | 0 | 100 | 0 | 0 |
| MAPK14 | 6 | 36068041 | sn | c.759T>C | p.H253H | . | . | . | . | rs2815805 | 0.04 | 0.016512 | 0.02100 | not listed | 95.59 | 4.41 | 0 | 97.17 | 2.83 | 0 |
| MAPK14 | 6 | 36075286 | ns | c.896C>T | p.A299V | NA | 5.61 | . | 5.494333 | . | . | . | . | not listed | 99.26 | 0.74 | 0 | 100 | 0 | 0 |
| MAPK3 | 16 | 30128224 | sn | c.1008G>A | p.P336P | . | . | . | . | rs1143695 | 0.0032 | 0.003023 | 0.00314 | not listed | 99.26 | 0.74 | 0 | 100 | 0 | 0 |
| MAPK3 | 16 | 30128580 | ns | c.802G>A | p.D268N | D | 5.35 | . | 2.862978 | . | . | . | . | not listed | 100 | 0 | 0 | 99.06 | 0.94 | 0 |
| MAPK3 | 16 | 30129377 | sn | c.651G>T | p.L217L | . | . | . | 2.257118 | rs139957276 | 0.0005 | 0.000465 | 0.00045 | not listed | 99.26 | 0.74 | 0 | 100 | 0 | 0 |
| MAPK3 | 16 | 30134507 | sn | c.24G>A | p.G8G | . | . | . | . | . | . | . | . | not listed | 100 | 0 | 0 | 98.11 | 1.89 | 0 |
| MAPK8 | 10 | 49609720 | ns | c.17G>A | p.R6H | T | 4.25 | . | 3.151268 | . | . | . | 0.00001 | not listed | 100 | 0 | 0 | 98.11 | 1.89 | 0 |
| MAPK8 | 10 | 49632183 | sn | c.669C>T | p.I223I | . | . | . | . | . | . | . | 0.00002 | not listed | 100 | 0 | 0 | 99.06 | 0.94 | 0 |
| MAPK8 | 10 | 49642974 | ns | c.1186G>A | p.V396I | T | 5.46 | . | 1.599793 | . | . | . | . | not listed | 99.26 | 0.74 | 0 | 100 | 0 | 0 |
| MAPK9 | 5 | 179665354 | sn | c.1110T>C | p.G370G | . | . | . | . | rs138473736 | . | 0.000465 | 0.00013 | not listed | 99.26 | 0.74 | 0 | 100 | 0 | 0 |
| MAPK9 | 5 | 179676062 | ns | c.527C>T | p.A176V | T | 4.11 | . | 2.462092 | . | . | . | . | not listed | 100 | 0 | 0 | 99.06 | 0.94 | 0 |
| NFKB1 | 4 | 103488139 | sp | c.256-5>TC | . | . | . | 0.25 | . | . | . | . | 0.00001 | not listed | 99.26 | 0.74 | 0 | 100 | 0 | 0 |
| NFKB1 | 4 | 103505961 | sn | c.1047C>T | p.Y349Y | . | . | . | . | rs4648039 | 0.01 | 0.024535 | 0.01500 | not listed | 88.97 | 11.03 | 0 | 97.17 | 2.83 | 0 |
| NFKB1 | 4 | 103514658 | sn | c.1140T>C | p.A380A | . | . | . | . | rs1609993 | 0.96 | 0.919186 | 0.94000 | not listed | 0.74 | 16.18 | 83.09 | 2.83 | 16.98 | 80.19 |
| NFKB1 | 4 | 103516146 | sp | c.1297+8>AG | . | . | . | 0.27 | . | . | . | . | 0.00006 | not listed | 99.26 | 0.74 | 0 | 100 | 0 | 0 |
| NFKB1 | 4 | 103517301 | ns | c.1304T>C | p.M435T | T | 4.6 | . | 1.092039 | . | . | . | 0.00004 | not listed | 99.26 | 0.74 | 0 | 100 | 0 | 0 |
| NFKB1 | 4 | 103518700 | ns | c.1516A>G | p.M506V | T | -4.82 | . | -0.21319 | rs4648072 | 0.01 | 0.008023 | 0.00973 | not listed | 97.79 | 2.21 | 0 | 99.06 | 0.94 | 0 |
| NFKB1 | 4 | 103527654 | ns | c.1751C>T | p.T584M | D | 4.59 | . | 5.152581 | . | . | . | . | not listed | 100 | 0 | 0 | 99.06 | 0.94 | 0 |
| NFKB1 | 4 | 103527745 | ns | c.1842G>T | p.L614F | T | 1.05 | . | 1.308065 | rs149211506 | 0.0027 | 0.003023 | 0.00194 | not listed | 98.53 | 1.47 | 0 | 100 | 0 | 0 |
| NFKB1 | 4 | 103534701 | sn | c.2709G>A | p.S903S | . | . | . | . | rs4648119 | . | 0.001047 | 0.00037 | not listed | 99.26 | 0.74 | 0 | 100 | 0 | 0 |
| NFKB1 | 4 | 103537672 | ns | c.2828C>A | p.T943N | T | 2 | . | 1.290152 | rs143882681 | . | 0.000581 | 0.00036 | not listed | 99.26 | 0.74 | 0 | 100 | 0 | 0 |
| NFKBIA | 14 | 35872068 | sp | c.548-3>CT | . | . | . | 0.18 | . | rs2233418 | 0.0018 | 0.008488 | 0.00652 | not listed | 98.53 | 1.47 | 0 | 99.06 | 0.94 | 0 |
| NFKBIA | 14 | 35872414 | sn | c.489G>A | p.L163L | . | . | . | . | . | . | . | . | not listed | 99.26 | 0.74 | 0 | 100 | 0 | 0 |
| NFKBIA | 14 | 35872926 | sn | c.306C>T | p.A102A | . | . | . | . | rs1050851 | 0.12 | 0.227791 | 0.16800 | not listed | 52.94 | 41.18 | 5.88 | 60.38 | 34.91 | 4.72 |
| NFKBIA | 14 | 35873770 | sn | c.81C>T | p.D27D | . | . | . | . | rs1957106 | 0.24 | 0.275911 | 0.27700 | not listed | 58.09 | 33.09 | 8.82 | 57.55 | 33.96 | 8.49 |
| NFKBIB | 19 | 39395836 | sp | c.28-6>CT | . | . | . | 0.67 | . | rs200550654 | . | . | 0.00002 | not listed | 99.26 | 0.74 | 0 | 100 | 0 | 0 |
| NFKBIB | 19 | 39396013 | ns | c.199C>T | p.R67C | T | -7.16 | . | 0.67979 | . | 0.0046 | 0.007286 | 0.00524 | not listed | 96.32 | 2.94 | 0.74 | 97.17 | 2.83 | 0 |
| NFKBIB | 19 | 39398188 | sn | c.600C>T | p.N200N | . | . | . | . | . | . | . | 0.00001 | not listed | 99.26 | 0.74 | 0 | 100 | 0 | 0 |
| NFKBIB | 19 | 39398201 | ns | c.613C>T | p.R205C | D | -0.188 | . | 2.135488 | rs187346322 | 0.0023 | 0.000818 | 0.00029 | not listed | 99.26 | 0.74 | 0 | 100 | 0 | 0 |
| NOD1 | 7 | 30487954 | ns | c.2245A>G | p.S749G | D | -2.15 | . | . | . | . | . | . | not listed | 99.26 | 0.74 | 0 | 100 | 0 | 0 |
| NOD1 | 7 | 30490919 | ns | c.2114G>A | p.R705Q | T | -3.79 | . | 0.13785 | rs144684378 | . | 0.002093 | 0.00085 | not listed | 99.26 | 0.74 | 0 | 100 | 0 | 0 |
| NOD1 | 7 | 30491123 | ns | c.1910G>A | p.R637H | T | 5.26 | . | 3.830579 | rs5743347 | 0.0018 | 0.002791 | 0.00138 | not listed | 99.26 | 0.74 | 0 | 100 | 0 | 0 |
| NOD1 | 7 | 30491143 | sn | c.1890C>T | p.G630G | . | . | . | . | . | . | . | . | not listed | 99.26 | 0.74 | 0 | 100 | 0 | 0 |
| NOD1 | 7 | 30491311 | sn | c.1722G>A | p.A574A | . | . | . | . | rs2075821 | 0.29 | 0.258953 | 0.27500 | not listed | 46.32 | 45.59 | 8.09 | 57.55 | 33.96 | 8.49 |
| NOD1 | 7 | 30491693 | ns | c.1340G>A | p.R447H | T | 3.22 | . | 3.31794 | rs2975634 | 0.02 | 0.000349 | 0.00001 | not listed | 99.26 | 0.74 | 0 | 100 | 0 | 0 |
| NOD1 | 7 | 30491837 | ns | c.1196G>A | p.R399Q | T | 1.4 | . | 3.429291 | rs141422065 | 0.0009 | 0.000465 | . | not listed | 100 | 0 | 0 | 99.06 | 0.94 | 0 |
| NOD1 | 7 | 30492086 | ns | c.947A>G | p.N316S | D | -3.6 | . | -1.23484 | . | . | . | 0.00005 | not listed | 100 | 0 | 0 | 99.06 | 0.94 | 0 |
| NOD1 | 7 | 30492142 | sn | c.891C>T | p.R297R | . | . | . | . | rs3020208 | 0.02 | 0.000349 | 0.00758 | not listed | 99.26 | 0.74 | 0 | 100 | 0 | 0 |
| NOD1 | 7 | 30492237 | ns | c.796G>A | p.E266K | D | 5.19 | . | 4.169 | rs2075820 | 0.3 | 0.245349 | 0.27700 | listed | 50 | 44.12 | 5.88 | 61.32 | 31.13 | 7.55 |
| NOD1 | 7 | 30492246 | ns | c.787C>A | p.R263R | . | . | . | 0.862199 | . | . | 0.000116 | 0.00002 | not listed | 99.26 | 0.74 | 0 | 100 | 0 | 0 |
| NOD1 | 7 | 30492550 | sn | c.483C>T | p.D161D | . | . | . | . | rs2235099 | 0.3 | 0.246395 | 0.27800 | not listed | 50 | 44.12 | 5.88 | 60.38 | 32.08 | 7.55 |
| NOD1 | 7 | 30492598 | sn | c.435G>T | p.L145L | . | . | . | . | rs5743340 | 0.0046 | 0.018256 | 0.01200 | not listed | 97.06 | 2.94 | 0 | 95.28 | 4.72 | 0 |
| NOD1 | 7 | 30494802 | sn | c.327C>T | p.F109F | . | . | . | . | . | . | . | 0.00534 | not listed | 100 | 0 | 0 | 99.06 | 0.94 | 0 |
| NOD1 | 7 | 30494866 | ns | c.263A>G | p.Y88C | T | 3.72 | . | 2.589497 | . | . | . | . | not listed | 99.26 | 0.74 | 0 | 100 | 0 | 0 |
| NOD1 | 7 | 30496382 | sn | c.156C>G | p.A52A | . | . | . | . | rs2075818 | 0.32 | 0.248488 | 0.28900 | not listed | 48.53 | 45.59 | 5.88 | 62.26 | 31.13 | 6.6 |
| NOD1 | 7 | 30496518 | ns | c.20G>A | p.S7N | T | 1.17 | . | 0.755481 | rs61757653 | . | . | 0.00026 | not listed | 99.26 | 0.74 | 0 | 100 | 0 | 0 |
| NOD2 | 16 | 50733392 | sp | c.74-7>TA | . | . | . | 1.83 | . | rs104895421 | 0.0014 | 0.001861 | 0.00001 | listed | 100 | 0 | 0 | 99.06 | 0.94 | 0 |
| NOD2 | 16 | 50733423 | ns | c.98C>A | p.A33D | T | 1.13 | . | 1.402571 | . | 0.000008 | . | 0.00001 | not listed | 99.26 | 0.74 | 0 | 100 | 0 | 0 |
| NOD2 | 16 | 50733661 | sn | c.336C>T | p.A112A | . | . | . | . | . | 0.00002 | . | 0.00003 | not listed | 100 | 0 | 0 | 99.06 | 0.94 | 0 |
| NOD2 | 16 | 50733785 | ns | c.460G>A | p.D154N | T | 0.958 | . | 0.410941 | rs146054564 | . | 0.002093 | 0.00064 | not listed | 100 | 0 | 0 | 99.06 | 0.94 | 0 |
| NOD2 | 16 | 50733859 | sn | c.534C>G | p.S178S | . | . | . | . | rs2067085 | 0.26 | 0.409302 | 0.33400 | not listed | 38.97 | 54.41 | 6.62 | 47.17 | 37.74 | 15.09 |
| NOD2 | 16 | 50741791 | ns | c.566C>T | p.T189M | T | 3.48 | . | 2.334887 | rs61755182 | 0.0014 | 0.004419 | 0.00259 | listed | 98.53 | 1.47 | 0 | 100 | 0 | 0 |
| NOD2 | 16 | 50741800 | ns | c.575C>T | p.A192V | D | 0.916 | . | 0.885309 | rs149071116 | 0.00004 | . | 0.00005 | not listed | 100 | 0 | 0 | 99.06 | 0.94 | 0 |
| NOD2 | 16 | 50741858 | sn | c.633C>T | p.A211A | . | . | . | . | rs5743269 | 0.0009 | 0.001744 | 0.00080 | not listed | 100 | 0 | 0 | 99.06 | 0.94 | 0 |
| NOD2 | 16 | 50744624 | ns | c.802C>T | p.P268S | T | -9.98 | . | -0.27189 | rs2066842 | 0.12 | 0.26907 | 0.18400 | listed | 42.65 | 47.06 | 10.29 | 55.66 | 34.91 | 9.43 |
| NOD2 | 16 | 50744688 | ns | c.866A>G | p.N289S | D | 4.56 | . | 0.444188 | rs5743271 | 0.01 | 0.006279 | 0.00425 | listed | 98.53 | 1.47 | 0 | 98.11 | 1.89 | 0 |
| NOD2 | 16 | 50744850 | ns | c.1028T>C | p.L343P | D | 5.4 | . | 0.517926 | . | . | 0.000116 | 0.00001 | not listed | 100 | 0 | 0 | 99.06 | 0.94 | 0 |
| NOD2 | 16 | 50745114 | ns | c.1292C>T | p.S431L | D | 3.64 | . | 0.851472 | rs104895431 | 0.0005 | 0.001395 | 0.00082 | listed | 99.26 | 0.74 | 0 | 100 | 0 | 0 |
| NOD2 | 16 | 50745199 | sn | c.1377C>T | p.R459R | . | . | . | . | rs2066843 | 0.13 | 0.270993 | 0.18500 | not listed | 41.91 | 47.79 | 10.29 | 50 | 39.62 | 10.38 |
| NOD2 | 16 | 50745316 | sn | c.1494A>G | p.E498E | . | . | . | . | . | . | . | . | not listed | 100 | 0 | 0 | 99.06 | 0.94 | 0 |
| NOD2 | 16 | 50745511 | sn | c.1689C>T | p.Y563Y | . | . | . | . | rs111608429 | 0.0005 | . | 0.00007 | not listed | 99.26 | 0.74 | 0 | 100 | 0 | 0 |
| NOD2 | 16 | 50745583 | sn | c.1761T>G | p.R587R | . | . | . | . | rs1861759 | 0.25 | 0.402558 | 0.32800 | not listed | 39.71 | 53.68 | 6.62 | 47.17 | 38.68 | 14.15 |
| NOD2 | 16 | 50745655 | sn | c.1833C>T | p.A611A | . | . | . | . | rs61736932 | 0.0046 | 0.010698 | 0.00983 | not listed | 98.53 | 1.47 | 0 | 100 | 0 | 0 |
| NOD2 | 16 | 50745751 | sn | c.1929C>T | p.L643L | . | . | . | . | . | 0.000008 | . | 0.00001 | not listed | 100 | 0 | 0 | 99.06 | 0.94 | 0 |
| NOD2 | 16 | 50745926 | ns | c.2104C>T | p.R702W | D | 2.42 | . | 1.736582 | rs2066844 | 0.02 | 0.043488 | 0.02300 | listed | 88.24 | 10.29 | 1.47 | 86.79 | 13.21 | 0 |
| NOD2 | 16 | 50745929 | ns | c.2107C>T | p.R703C | D | 2.89 | . | 1.788325 | rs5743277 | 0.0023 | 0.006977 | 0.00002 | listed | 97.79 | 2.21 | 0 | 100 | 0 | 0 |
| NOD2 | 16 | 50745960 | ns | c.2138G>A | p.R713H | T | 4.13 | 0.75 | 2.225724 | rs104895483 | . | 0.000233 | 0.00034 | listed | 98.53 | 1.47 | 0 | 100 | 0 | 0 |
| NOD2 | 16 | 50746086 | ns | c.2264C>T | p.A755V | D | 5.12 | . | 1.225314 | rs61747625 | 0.0005 | 0.004651 | 0.00231 | listed | 98.53 | 1.47 | 0 | 100 | 0 | 0 |
| NOD2 | 16 | 50746199 | ns | c.2377G>A | p.V793M | D | 3.51 | . | 1.544959 | rs104895444 | 0.0005 | 0.001628 | 0.00105 | listed | 99.26 | 0.74 | 0 | 98.11 | 1.89 | 0 |
| NOD2 | 16 | 50746228 | sn | c.2406G>T | p.V802V | . | . | . | 1.92838 | rs104895495 | . | 0.00186 | 0.00196 | not listed | 98.53 | 1.47 | 0 | 99.06 | 0.94 | 0 |
| NOD2 | 16 | 50746291 | sp | c.2462+7>GT | . | . | . | 0.83 | . | rs202111813 | 0.0005 | 0.000581 | 0.00016 | not listed | 99.26 | 0.74 | 0 | 100 | 0 | 0 |
| NOD2 | 16 | 50750842 | ns | c.2587A>G | p.M863V | T | -9.48 | . | 0.558526 | rs104895447 | . | 0.00186 | 0.00120 | listed | 99.26 | 0.74 | 0 | 100 | 0 | 0 |
| NOD2 | 16 | 50756540 | ns | c.2722G>C | p.G908R | D | 5.56 | . | 5.54325 | rs2066845 | 0.01 | 0.014535 | 0.00992 | listed | 96.32 | 3.68 | 0 | 96.23 | 3.77 | 0 |
| NOD2 | 16 | 50756571 | ns | c.2753C>A | p.A918D | D | 5.56 | . | 5.735298 | rs104895452 | 0.0009 | 0.000814 | 0.00040 | listed | 100 | 0 | 0 | 99.06 | 0.94 | 0 |
| NOD2 | 16 | 50757276 | ns | c.2863G>A | p.V955I | T | -9.14 | . | -0.87026 | rs5743291 | 0.05 | 0.096047 | 0.06100 | listed | 83.09 | 16.91 | 0 | 81.13 | 18.87 | 0 |
| NOD2 | 16 | 50759405 | ns | c.288A8>G | p.E963G | T | 5.29 | . | 4.950708 | . | . | . | . | not listed | 99.26 | 0.74 | 0 | 100 | 0 | 0 |
| NOD2 | 16 | 50763778 | fr | c.3019dupC | p.L1007fs | . | . | . | . | rs2066847 | 0.006 | . | . | listed | 89.71 | 8.82 | 1.47 | 99.06 | 0.94 | 0 |
| RELA | 11 | 65422007 | ns | c.1498A>G | p.I500V | D | 3.39 | 1.83 | 2.237479 | . | . | . | . | not listed | 99.26 | 0.74 | 0 | 100 | 0 | 0 |
| RELA | 11 | 65425764 | ns | c.871G>A | p.D291N | T | 4.76 | . | 3.374902 | rs61759893 | 0.0046 | 0.004655 | 0.00346 | not listed | 98.53 | 1.47 | 0 | 99.06 | 0.94 | 0 |
| RELA | 11 | 65425804 | sn | c.831C>T | p.D277D | . | . | . | . | rs147357241 | . | 0.000233 | 0.00016 | not listed | 99.26 | 0.74 | 0 | 100 | 0 | 0 |
| RELA | 11 | 65427183 | ns | c.513G>T | p.R171S | T | 4.36 | . | 3.419675 | . | . | . | 0.00001 | not listed | 100 | 0 | 0 | 99.06 | 0.94 | 0 |
| RIPK2 | 8 | 90770315 | sn | c.27C>T | p.A9A | . | . | . | . | rs2293809 | 0.09 | 0.031789 | 0.06200 | not listed | 92.65 | 6.62 | 0.74 | 94.34 | 5.66 | 0 |
| RIPK2 | 8 | 90784979 | ns | c.776T>C | p.I259T | T | 4.45 | . | 3.648531 | rs2230801 | 0.08 | 0.07907 | 0.06800 | not listed | 84.56 | 15.44 | 0 | 78.3 | 21.7 | 0 |
| RIPK2 | 8 | 90801670 | sn | c.1245T>C | p.S415S | . | . | . | . | rs56109184 | 0.0005 | 0.000698 | 0.00025 | not listed | 100 | 0 | 0 | 99.06 | 0.94 | 0 |
| RIPK2 | 8 | 90802491 | sn | c.1470A>G | p.L490L | . | . | . | . | rs16900617 | 0.08 | 0.002558 | 0.02400 | not listed | 99.26 | 0.74 | 0 | 100 | 0 | 0 |
| RIPK2 | 8 | 90802611 | sn | c.1590A>G | p.P530P | . | . | . | . | rs186397742 | 0.0009 | . | 0.00012 | not listed | 100 | 0 | 0 | 99.06 | 0.94 | 0 |
| SUGT1 | 13 | 53231709 | ns | c.139T>A | p.Y47N | D | 4.12 | . | . | . | . | . | . | not listed | 99.26 | 0.74 | 0 | 100 | 0 | 0 |
| SUGT1 | 13 | 53239767 | sp | c.519-5>AT | . | . | . | 1.07 | . | . | . | . | 0.00001 | not listed | 100 | 0 | 0 | 98.11 | 1.89 | 0 |
| SUGT1 | 13 | 53240958 | ns | c.627G>T | p.L209F | D | 2.93 | . | 2.604733 | rs61756205 | 0.0018 | 0.005119 | 0.00252 | not listed | 100 | 0 | 0 | 99.06 | 0.94 | 0 |
| SUGT1 | 13 | 53254116 | ns | c.822G>T | p.K274N | T | 3.92 | . | 2.817749 | rs202155148 | . | 0.000116 | 0.00008 | not listed | 99.26 | 0.74 | 0 | 99.06 | 0.94 | 0 |
| SUGT1 | 13 | 53254296 | sp | c.996+6>AG | . | . | . | 0.75 | . | rs7986540 | 0.97 | 0.946395 | 0.95200 | not listed | 1.47 | 7.35 | 91.18 | 4.72 | 11.32 | 83.96 |
| SUGT1 | 13 | 53261936 | nonfd | c.1069_1071del | p.357_357del | . | . | . | . | . | . | . | . | not listed | 100 | 0 | 0 | 99.06 | 0.94 | 0 |
| TAB1 | 22 | 39795831 | sn | c.24G>A | p.L8L | . | . | . | . | . | . | . | 0.00003 | not listed | 99.26 | 0.74 | 0 | 100 | 0 | 0 |
| TAB1 | 22 | 39814746 | ns | c.560G>A | p.R187H | D | 4.45 | . | 6.166536 | rs140879164 | 0.0009 | 0.000233 | 0.00058 | not listed | 99.26 | 0.74 | 0 | 100 | 0 | 0 |
| TAB1 | 22 | 39814802 | ns | c.616G>A | p.D206N | T | 4.45 | . | 4.232386 | rs148869940 | . | 0.000698 | 0.00012 | not listed | 99.26 | 0.74 | 0 | 100 | 0 | 0 |
| TAB1 | 22 | 39826049 | ns | c.1337C>T | p.T446I | T | 4.67 | . | 5.706612 | rs118074217 | 0.0005 | 0.001163 | 0.00055 | not listed | 100 | 0 | 0 | 99.06 | 0.94 | 0 |
| TAB1 | 22 | 39826137 | sn | c.1425C>T | p.D475D | . | . | . | . | rs147601362 | 0.0009 | 0.001395 | 0.00094 | not listed | 100 | 0 | 0 | 99.06 | 0.94 | 0 |
| TAB1 | 22 | 39832516 | sn | c.1329C>T | p.S443S | . | . | . | . | . | . | 0.000116 | 0.00004 | not listed | 99.26 | 0.74 | 0 | 100 | 0 | 0 |
| TAB2 | 6 | 149699333 | sn | c.282A>G | p.G94G | . | . | . | . | rs13215304 | 0.02 | 0.031163 | 0.02000 | not listed | 100 | 0 | 0 | 100 | 0 | 0 |
| TAB2 | 6 | 149699483 | sn | c.432T>C | p.S144S | . | . | . | . | . | . | . | . | not listed | 100 | 0 | 0 | 100 | 0 | 0 |
| TAB2 | 6 | 149699483 | sn | c.432T>C | p.S144S | . | . | . | . | . | . | . | . | not listed | 100 | 0 | 0 | 100 | 0 | 0 |
| TAB2 | 6 | 149700128 | sn | c.1077C>T | p.T359T | . | . | . | . | rs138731123 | . | . | 0.00019 | not listed | 100 | 0 | 0 | 100 | 0 | 0 |
| TAB2 | 6 | 149700491 | sn | c.1440G>A | p.V480V | . | . | . | . | rs3734296 | 0.21 | 0.105698 | 0.17100 | not listed | 100 | 0 | 0 | 100 | 0 | 0 |
| TAB2 | 6 | 149730846 | sn | c.2073G>A | p.R691R | . | . | . | . | rs652921 | 0.21 | 0.105698 | 0.17100 | not listed | 100 | 0 | 0 | 100 | 0 | 0 |
| TAB3 | X | 30849697 | sp | c.1991-5>CT | . | . | . | 0.12 | . | rs202074143 | . | 0.000595 | 0.00047 | not listed | 100 | 0 | 0 | 99.06 | 0.94 | 0 |
| TAB3 | X | 30870971 | ns | c.1634C>G | p.S545C | T | 4.31 | . | 2.179871 | . | . | . | 0.00090 | not listed | 100 | 0 | 0 | 99.06 | 0 | 0.94 |
| TAB3 | X | 30873039 | ns | c.743C>T | p.T248M | D | 2.53 | . | 0.808718 | . | . | . | 0.00021 | not listed | 99.26 | 0 | 0.74 | 100 | 0 | 0 |
| TAB3 | X | 30873245 | sn | c.537G>A | p.P179P | . | . | . | . | rs146319957 | 0.0018 | 0.00431 | 0.00340 | not listed | 98.53 | 1.47 | 0 | 100 | 0 | 0 |
| TNF | 6 | 31544562 | ns | c.251C>T | p.P84L | T | 0.92 | . | 1.458155 | rs4645843 | . | 0.002953 | 0.00235 | not listed | 98.53 | 1.47 | 0 | 100 | 0 | 0 |
| TNFAIP3 | 6 | 138196066 | ns | c.380T>G | p.F127C | T | 0.836 | . | 1.667118 | rs2230926 | 0.12 | 0.032093 | 0.06100 | listed | 100 | 0 | 0 | 100 | 0 | 0 |
| TNFAIP3 | 6 | 138196817 | sp | c.487-8>CG | . | . | . | 0.45 | . | rs5029947 | 0.02 | 0.00093 | 0.00562 | not listed | 100 | 0 | 0 | 100 | 0 | 0 |
| TNFAIP3 | 6 | 138199644 | sn | c.1062G>A | p.K354K | . | . | . | . | . | . | 0.000233 | 0.00018 | not listed | 100 | 0 | 0 | 100 | 0 | 0 |
| TNFAIP3 | 6 | 138199950 | sn | c.1368G>C | p.G456G | . | . | . | . | rs201600532 | 0.0005 | . | 0.00012 | not listed | 100 | 0 | 0 | 100 | 0 | 0 |
| TNFAIP3 | 6 | 138201240 | ns | c.1939T>C | p.S647P | T | 0.362 | . | 1.04551 | rs142253225 | 0.0009 | 0.002791 | 0.00188 | not listed | 100 | 0 | 0 | 100 | 0 | 0 |
| TNFAIP3 | 6 | 138202258 | sn | c.2175G>A | p.L725L | . | . | . | . | rs140354477 | 0.0018 | 0.002442 | 0.00180 | not listed | 100 | 0 | 0 | 100 | 0 | 0 |
| TNFAIP3 | 6 | 138202378 | sn | c.2295C>T | p.P765P | . | . | . | . | rs5029956 | 0.02 | 0.000465 | 0.00827 | not listed | 100 | 0 | 0 | 100 | 0 | 0 |
| TRAF6 | 11 | 36514122 | ns | c.735T>A | p.S245R | T | 3.02 | . | 2.144458 | . | . | 0.000116 | 0.00002 | not listed | 99.26 | 0.74 | 0 | 100 | 0 | 0 |
| TRIP6 | 7 | 100465128 | sn | c.9G>A | p.G3G | . | . | . | . | . | . | . | . | not listed | 100 | 0 | 0 | 99.06 | 0.94 | 0 |
| TRIP6 | 7 | 100465747 | ns | c.255G>T | p.R85S | T | -5.88 | . | 0.195055 | rs139351872 | 0.0014 | 0.000698 | . | not listed | 99.26 | 0.74 | 0 | 100 | 0 | 0 |
| TRIP6 | 7 | 100465807 | sn | c.315C>T | p.A105A | . | . | . | . | rs144580285 | 0.0014 | 0.003605 | 0.00230 | not listed | 99.26 | 0.74 | 0 | 100 | 0 | 0 |
| TRIP6 | 7 | 100465824 | ns | c.332G>A | p.R111Q | T | 3.13 | . | 1.472456 | rs2437100 | 0.01 | 0.020465 | 0.01600 | not listed | 96.32 | 3.68 | 0 | 95.28 | 4.72 | 0 |
| TRIP6 | 7 | 100466176 | sn | c.423C>T | p.A141A | . | . | 1.07 | . | . | . | . | 0.00001 | not listed | 99.26 | 0.74 | 0 | 100 | 0 | 0 |
| TRIP6 | 7 | 100466441 | ns | c.688G>A | p.V230I | T | 4.66 | . | 1.751354 | rs2075756 | 0.28 | 0.269014 | 0.28500 | not listed | 55.15 | 38.97 | 5.88 | 66.98 | 27.36 | 5.66 |
| TRIP6 | 7 | 100466457 | ns | c.704G>C | p.G235A | T | 2.36 | . | 1.995768 | . | . | . | 0.00001 | not listed | 100 | 0 | 0 | 99.06 | 0.94 | 0 |
| TRIP6 | 7 | 100468284 | sn | c.918a>G | p.V306V | . | . | . | 2.457742 | rs1054391 | 0.57 | 0.523372 | 0.56200 | not listed | 25.74 | 49.26 | 25 | 27.36 | 47.17 | 25.47 |
| TRIP6 | 7 | 100468345 | ns | c.979T>C | p.Y327H | D | 5.47 | . | 2.389748 | . | . | . | . | not listed | 98.53 | 1.47 | 0 | 100 | 0 | 0 |
| TRIP6 | 7 | 100469219 | ns | c.1054C>T | p.R352W | D | 3.04 | . | 2.48622 | . | . | . | 0.00001 | not listed | 100 | 0 | 0 | 99.06 | 0.94 | 0 |
| TRIP6 | 7 | 100469223 | ns | c.1058C>T | p.A353V | D | 4.21 | . | 2.699637 | rs147492293 | . | 0.000349 | 0.00012 | not listed | 99.26 | 0.74 | 0 | 100 | 0 | 0 |
| XIAP | X | 123034511 | ns | c.1268A>C | p.Q423P | T | 4.26 | . | 0.733377 | rs5956583 | 0.261 | 0.33 | 0.33000 | listed | 52.21 | 24.26 | 23.53 | 60.38 | 16.04 | 23.58 |
| XIAP | X | 123040945 | ns | c.1408A>T | p.T470S | T | 4.11 | . | 1.624093 | rs143165174 | . | 0.000595 | 0.00048 | listed | 99.26 | 0 | 0.74 | 100 | 0 | 0 |

ns, non-synonymous; sn, synonymous; fi, frameshift insertion, fd, frameshift deletion; sp, splicing; nfi, non-frameshift insertion; nfd, non-frameshift deletion,sp, splicing

B, benign; C, Conservative; D, deleterious; MC, moderately Conservative; MR, moderately Radical; NR, not reported; P, possibly damaging; R

**Table S3 Joint variant test (SKAT-O) result for the NOD2 gene in which variations was found across the entire discovery cohort.**

| Gene | Chromosome | Total number of samples (136 cases; 106 controls) | bp position (hg19) | Fraction of individuals who carry rare variants under the MAF thresholds (MAF < 0.05)* | Number of all variants defined in the group file | Number of variant defined as rare (MAF < 0.05)* | P-value unadjusted |
| --- | --- | --- | --- | --- | --- | --- | --- |
| *NOD2* | 16 | 242 | 50745926-50763778 | 0.098765 | 3^+^ | 2 | 0.016861 |
|  |  |  | 50733392-50759405 | 0.13992 | 28• | 23 | 0.5441 |

+ gene based test conducted on only the three NOD2 biomarkers: Arg702Trp, Gly908Arg and Leu1007fsinsC

• gene based test conducted excluding three NOD2 biomarkers: Arg702Trp, Gly908Arg and Leu1007fsinsC

* These variants received different weights in the SKAT-O joint test. Genes are ordered by p-value
